# Factors influencing health behavior changes in people with early multiple sclerosis: process evaluation of the multicenter randomized controlled POWER@MS1 trial

**DOI:** 10.3389/fneur.2025.1635872

**Published:** 2025-10-27

**Authors:** Barbara von Glasenapp, Nicole Krause, Carlotta Derad, Karin Riemann-Lorenz, Björn Meyer, Markus van de Loo, Herbert Temmes, Stefan Gold, Christoph Heesen

**Affiliations:** ^1^Institute of Neuroimmunology and Multiple Sclerosis, University Medical Center Hamburg-Eppendorf, Hamburg, Germany; ^2^Department of Medical Statistics, University Medical Centre Göttingen, Göttingen, Germany; ^3^Research and Development Department, GAIA Group, Hamburg, Germany; ^4^German Multiple Sclerosis Society, Federal Association, Hannover, Germany; ^5^Department of Psychiatry (CBF), Charité Universitätsmedizin Berlin, Berlin, Germany

**Keywords:** multiple sclerosis, mixed methods, digital health application, health behavior change, process evaluation

## Abstract

**Background:**

Health behavior changes, i.e., optimizations of physical activity, diet, sleep and stress management, are increasingly considered as modifiers of prognostic risk in multiple sclerosis (MS). A personalized digital lifestyle management application (“levidex”), designed to support people with MS (pwMS) in coping with their diagnosis and adopting healthier behaviors, was evaluated against an active psychoeducational control program (“dexilev”) in a randomized controlled trial (RCT; “POWER@MS1”).

**Objectives:**

This study evaluates the POWER@MS1 trial, focusing on the processes and organizational aspects of the study. Specifically, it seeks to (1) identify the contextual factors that influence behavior change in pwMS and (2) assess how the intervention and study design were perceived by pwMS and involved health care professionals (HCPs; neurologists, study nurses, radiologists).

**Methods:**

A mixed methods approach was applied. During the study period questionnaire data were collected from all trial participants (*n* = 234) and HCPs (*n* = 91) and were analyzed quantitatively. After the RCT ended, semi-structured interviews were conducted with 15 HCPs and 16 pwMS. Participants were selected according to the maximum variation sampling. Data was analyzed thematically.

**Results:**

Quantitative trial data revealed that the levidex group significantly agreed more to behavioral changes after 3 months [levidex (6.65); dexilev (5.23), *p* < 0.001]. Improvements in diet, physical activity and stress management were reported. PwMS considered evidence-based information, meditation instructions and self-monitoring tools embedded in levidex as particularly helpful. In the interviews, they reported close monitoring through regular clinical visits as reassuring after MS diagnosis. A healthy lifestyle was considered an important component of MS treatment by both HCPs and pwMS. Both perceived levidex as a useful addition to standard care, but reported a need for additional personal consultation.

**Conclusion:**

Health behavior change was rated as an important component of MS treatment. A digital application was perceived to be beneficial for the facilitation of relevant behavior change.

## 1 Introduction

Multiple sclerosis (MS) is an immunological and neurodegenerative disease that can lead to a wide range of symptoms, such as fatigue, cognitive impairment and muscle weakness. In Germany, around 280,000 people are affected by MS (1). The exact pathogenesis of the disease is not yet fully understood; however, it is assumed that both genetic and environmental factors contribute to disease onset (2). While immunotherapies show reduction of inflammatory activity in MS, lifestyle factors seem to also impact on the disease progression (3–6). Although there is a growing consensus that health behaviors (especially diet, exercise and smoking) are important in the treatment of MS, counseling on disease-modifying drugs is the key focus in most medical encounters (4–7). Digital health applications are increasingly developed to manage well-being and symptoms, such as MS-related fatigue and depression (8). People with MS (pwMS) show a high affinity to digital tools, and digital behavioral interventions have already shown beneficial effects in managing MS symptoms (9). A digital health intervention termed “levidex” was developed to provide pwMS with evidence-based information in early stages of the disease and to support relevant health behavior changes (HBC), such as adopting a healthier diet, a physically active daily routine, and improved stress and sleep management habits. POWER@MS1 was a multicenter randomized controlled trial (RCT) to compare the effects of levidex with a psychoeducational control program (“dexilev”) on inflammatory disease activity and clinical outcomes, including health behaviors (10). The RCT was accompanied by a process evaluation. As especially in behavioral interventions attrition and study outcome lack subjective and qualitative data and the perspective of health care professionals (HCPs) is mostly not included, process evaluations are highly meaningful and recommended (11). The actual study aimed to evaluate the implementation of the study and to understand the perception of participating HCPs, such as study nurses, neurologists and radiologists, as well as pwMS, regarding digital health applications (DHA) in general and their potential for MS management.

## 2 Methods

**Figure 1:**
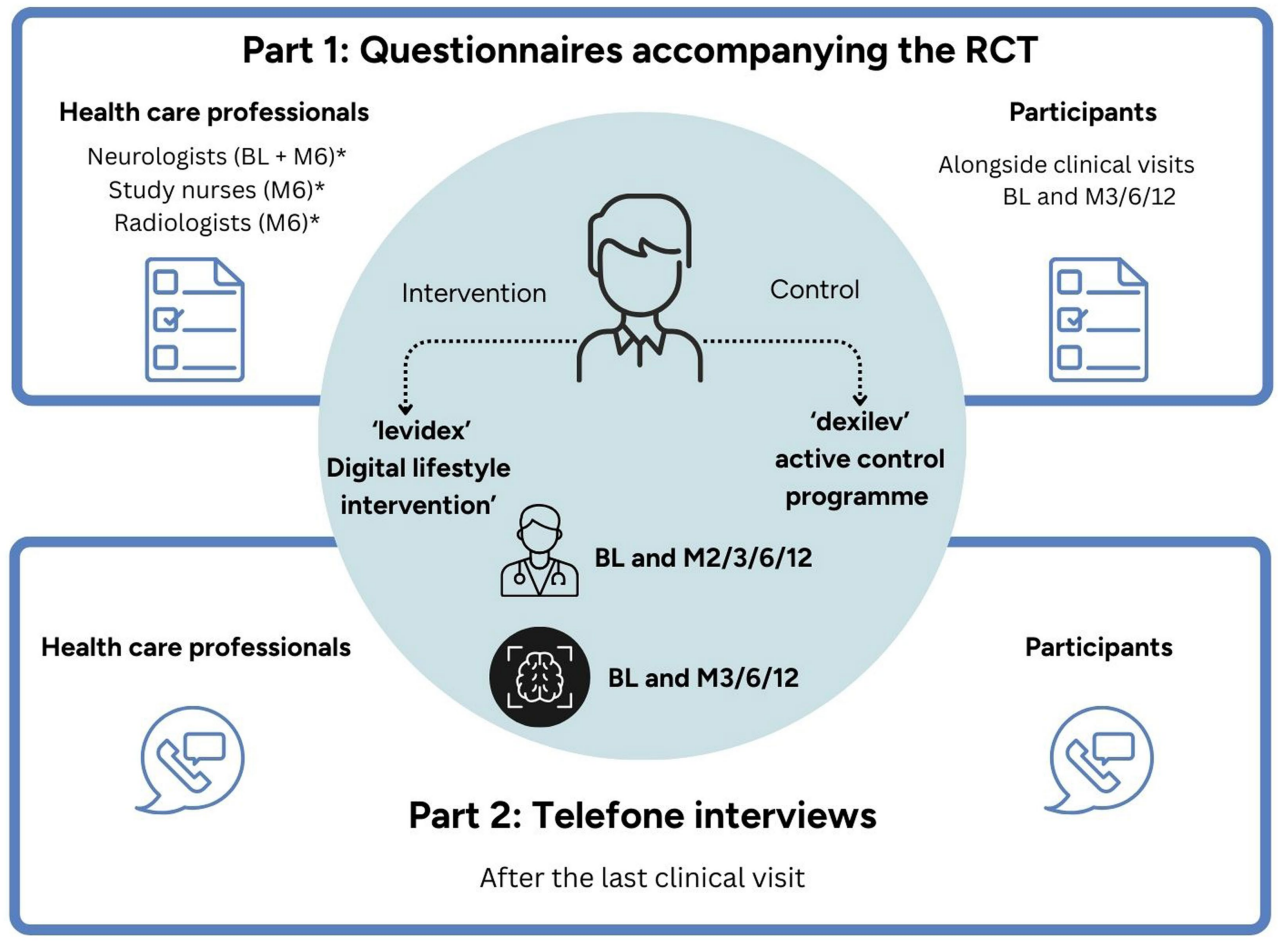
Process evaluation: time points and key components. BL, baseline; M2/M3/M6/M12, month 2/3/6/12 after randomization; RCT, randomized controlled trial. *Indicates time points after enrollment. Elements highlighted in light blue correspond to the main RCT; elements outlined in dark blue represent the process evaluation.

A study protocol for the RCT, including the implementation of the process, evaluation has been published elsewhere (12). Evaluation was carried out according to the Medical Research Council framework for the development and evaluation of complex interventions using a mixed methods approach involving HCPs and pwMS who participated in the RCT (13). During the POWER@MS1 study, the intervention group (IG) had 12-month access to levidex, a personalized digital lifestyle management application covering different areas of health behavior. In addition to a series of interactive “simulated conversations” on relevant lifestyle topics (e.g., diet, exercise, stress management), the program also includes accompanying materials, such as audio exercises, weekly health check-ins and worksheets. The control group (CG) received access to *dexilev*, a non-personalized psychoeducational program that provided evidence-based information on the same health behavior topics as levidex, but without any additional materials or interactive features. Next to the evaluation of the participants, the experiences of the HCPs who accompanied pwMS during the intervention in routine care were additionally evaluated. Quantitative data for process evaluation was collected using self-developed questionnaires (PEQ) at different time points throughout the study (see Figure 1). Telephone interviews were conducted after the end of the study. For each section, first the quantitative part will be reported and is followed by the qualitative part. First, the data of the HCPs will be presented, and then the data of the pwMS. For a detailed list of the main objectives of the process evaluation (see Supplementary material A).

### 2.1 Sampling

Questionnaires were provided to all neurologists, study nurses and radiologists at all 20 participating study centers across Germany. HCPs were contacted via mail by the study team at UKE according to the time point they had to fill out their questionnaires.

For the qualitative part, HCPs were selected based on criteria such as the type of institution (academic hospital, community clinic or private practice), maximal and minimal recruiters (inclusion of pwMS) and based on the information of the PEQ to ensure a broad range of responses.

For the quantitative part of the process evaluation, PwMS got handed out PEQ alongside their clinical visits by the study centers. PwMS of both treatment groups were invited following a maximum variation sampling strategy to participate in the qualitative part of the evaluation. Age, sex, assignment to the IG or CG, application usage behavior (i.e., total days with activity, module completion), and self-reported HBC were taken into account for selection.

### 2.2 Data collection

Questionnaires were completed paper-based or digitally through a platform administered by the German MS registry based on secuTrial® run by the University of Göttingen. HCPs were asked to fill out PEQ at baseline (neurologists) and 6 months after the inclusion of the first participant (neurologists, study nurses and radiologists). To collect qualitative data, individual semi-structured interview guides were developed for each profession. HCP interviews were conducted between May and July 2023.

For quantitative data, pwMS were assessed at baseline, month 3, month 6 and month 12 after randomization (see Supplementary material B). For each group, individual semi-structured interview guides were developed. PwMS interviews were held between December 2023 and January 2024. All interview guides are provided in Supplementary material C.

### 2.3 Data processing

Questionnaires were completed on paper and transferred to secuTrial® by the study team. The data was exported from the data platform and analyzed using Excel (Microsoft 365) as well as R version 4.3.1 (R Foundation for Statistical Computing, Vienna, Austria). The interviews were recorded and transcribed using the software f4x by dr. dressing & pehl GmbH, Marburg, Germany. Transcripts were quality checked and analyzed using MAXQDA (Version 2022, VERBI Software GmbH, Berlin, Germany).

### 2.4 Data analysis

For quantitative data, mean values and standard deviation (SD) were determined. Characteristics of participants are reported as median and range. T-tests were performed to analyze group differences in metric variables. The chi-square test was used for categorical variables with multiple response options. For analysis of the impact of key baseline demographic factors (i.e., sex, age, education), analyses of variance (ANOVA) based on a linear model were performed. The model included group, the baseline variable, and their interaction.

Differences between IG and CG were reported as pairwise contrasts of estimated marginal means derived from the linear model with 95% confidence interval (CI) and *p*-value for interaction, and are visualized within a forest plot. In case of a significant finding, Spearman correlation was performed. A Spearman correlation coefficient <0.1 is considered negligible, from 0.1 to <0.3 weak, 0.3 to <0.5 moderate and above 0.5 strong.

Interviews were coded independently by two researchers (BvG and NK). The data was analyzed according to the six steps of Braun and Clark (14). First, coders familiarized themselves with the data by reading the transcripts. Second, NK developed an initial coding system based on the transcripts and interview guides. Individual code systems were developed for neurologists, study nurses, radiologists and pwMS. Third, NK and BvG discussed the initial categories and consented a coding system. The codes were developed inductively and deductively. Fourth, categories were reviewed after coding of the first three transcripts. It was discussed whether the code system was working and, if necessary, extended. Fifth, after agreeing on a final code set, the data were coded independently by NK and BvG and checked for consistency between both coders. In case of discrepancies, the text passages in question were discussed and a categorization was agreed.

### 2.5 Participant characteristics

#### 2.5.1 Characteristics of health care professionals

Forty-three neurologists at baseline and 41 neurologists, 28 study nurses, and 20 radiologists at month 6 completed the PEQ. All questionnaires were fully completed. Thirty HCPs (12 neurologists, 10 study nurses and eight radiologists) were invited to participate in the interview. Six interview requests were declined due to lack of time, holidays or end of employment at the participating center. Six requests remained unanswered. Nine neurologists, five study nurses and four radiologists from 13 of the study centers agreed to be interviewed. Of the neurologists interviewed, four were employed in academic hospitals, three in community clinics and two in private practices. Based on their own assessment, four of the neurologists stated that they had excellent MS knowledge, while four rated their knowledge as high. Three of the study nurses also rated their knowledge of MS as high (see Table 1).

**Table 1:** Sample characteristics of health care professionals.

Variable	n (%) or median (range)					
	Interview participants			Questionnaires		
	Neurologists *n* = 9	Nurses *n* = 5	Radiologists *n* = 4	Neurologists (BL) *n* = 43	Nurses (M6) *n* = 28	Radiologists (M6) *n* = 20
Sex; *n (%)*						
Female	3 (30)	5 (100)	3 (75)	22 (51)	28 (100)	6 (30)
Male	6 (60)	0 (0)	1 (25)	21 (49)	0 (0)	13 (65)
Divers	0 (0)	0 (0)	0 (0)	0 (0)	0 (0)	1 (5)
Age in years*; M (range)*	47 (34–60)	52 (42–57)	n.d.	37 (24–60)	39.5 (24–65)	48 (35–65)
Type of study center; *n(%)*						
Academic hospital	4 (44)	1 (20)	1 (25)	27 (62.7)^a^	15(53.6)^b^	7 (35)
Community clinic	3 (33)	1 (20)	2 (50)	11 (25.5)	8(28.6)	8 (40)
Private practice	2 (22)	3 (60)	1 (25)	5 (11.6)	5 (17.8)	5 (25)
MS experience in years*; M (range)*	16 (5–27)	10 (4–27)	n.d.	9.7 (2–29)	8,7 (1–27)	16 (8–37)
Self-reported MS knowledge; *n (%)*						
Excellent	4 (44)	0 (0)	1 (25)	10 (23)	3 (10)	5 (25)
High	4 (44)	3 (60)	2 (75)	24 (56)	12 (42)	13 (65)
Moderate	1 (11)	2 (40)	1 (25)	8 (19)	13 (46)	2 (10)
Limited	0 (0)	0 (0)	0 (0)	1 (0)	0 (0)	0 (0)

BL, Baseline; MS, Multiple Sclerosis; M6, Month 6 after Baseline; UKE, University Medical Center Hamburg-Eppendorf; n.d., no data.

a 12 neurologists from the University Medical Center Hamburg-Eppendorf.

b 2 study nurses from the University Medical Center Hamburg-Eppendorf.

#### 2.5.2 Characteristics of participating people with MS

The PEQ were completed by participating pwMS alongside the outcome inventories for clinical study visits. For detailed characterizations, see the results of the main trial (10).

In total, 30 pwMS were invited to a telephone interview. We did not hear back from 12 of those contacted. One pwMS was on holiday and one only responded after the end of the interview period.

It was not possible to recruit any pwMS who dropped out of the study early for an interview. Sixteen pwMS, 12 of whom were female, with a median age of 42.5 years (23–64), took part in the interviews, 11 from the IG and five from the CG. Detailed sample characteristics can be found in Table 2. The detailed analysis of the qualitative interviews is provided in Supplementary material D.

**Table 2:** Characteristics of participating people with MS.

Sample participating people with MS	Questionnaire cohort (BL) *n* = 234	Interview cohort *n* = 16
Sex, *n (%)*		
Female	184 (78.6)	12 (75)
Male	50 (21.4)	4 (25)
Age in years, *M (range)*	34.5 (18–61)	42.5 (23–64)
Intervention or control group, *n (%)*		
levidex (intervention)	115 (49.1)	11 (68.8)
dexilev (control)	119 (50.9)	5 (31.3)
EDSS, *M (range)*		
levidex	1.0 (0.0–4.0)	1.0 (0.0–3.5)
dexilev	1.5 (0.0–4.0)	2.0 (0.0–3.5)
Years of education^a^, *n (%)*		
Less than 12 years	75 (33.2)	4 (25)
12 years or more	151 (66.8)	12 (75)
User activity online program^b^ *M (range)*		
levidex	17.5 (1–256)	17 (9–256)
dexilev	11 (1–24)	13 (10–13)

BL, baseline; EDSS, Expanded Disability Status Scale; IQR, interquartile range; M, Median; n.d, no data; SD, standard deviation.

a Missing values, *n* = 8.

b Total days with login of pwMS on the platform (levidex *n* = 1 and dexilev *n* = 5 without registration).

## 3 Results

Findings are displayed based on topics, combining quantitative and qualitative data. To ensure a structured reporting of results, both quantitative and qualitative findings are presented based on three main categories derived from the qualitative analysis: “Relevance of health behavior and immunotherapy,” “Perception of digital health applications” and “General feedback on POWER@MS1.”

### 3.1 Relevance of health behavior and immunotherapy

#### 3.1.1 Perceived relevance of health behaviors by HCPs

In order to inquire about attitudes toward disease-modifying medication, the neurologists were asked whether in their opinion, all pwMS should start an immunotherapy treatment after diagnosis, which six agreed to, 16 somewhat agreed, 13 somewhat disagreed to and eight disagreed to. An open question in the PEQ evaluated under which circumstances a “watch-and-wait” approach would be appropriate. (Very) low lesion burden, clinically (very) low disease activity (few/mild relapses), good recovery of relapses, as well as patient concerns about drug-related side effects, the decision of a well-informed patient against a therapy, advanced age and the patients desire to have children were reported. When asked whether they believe that health behavior influences disease activity or progression, 35 out of 43 neurologists (81.4%) agreed, seven somewhat agreed, and one somewhat disagreed. In response to the statement “If a patient is well informed, I can accept their decision, even if I would recommend something else,” 30 neurologists agreed, 10 somewhat agreed while three somewhat disagreed or disagreed.

The interviews confirmed the aforementioned statements regarding the reasons justifying a watch-and-wait approach. When asked about the clinical relevance of lifestyle factors in relation to disease progression all HCPs considered health behavior as a relevant factor in MS treatment. In particular, the positive effect of moderate exercise and physical activity as well as the negative consequences of smoking were mentioned here.

*“But to really do something about this long-term progression, I think lifestyle factors are the most important. To be honest, we don't have any medication, but lifestyle will probably make a big difference here."(N_11)*

Even among the neurologists who perceived lifestyle factors as a very important component in MS treatment, HBCs were mentioned as an additive treatment approach rather than as an alternative to immunotherapy. Patients’ need for MS-related lifestyle information was rated as very high by both neurologists and study nurses, especially in the first year after MS diagnosis.

*“[…] but also that it is made clear to patients from the outset that they are not helpless, but that there are massive possibilities, especially in the area of lifestyle factors. To influence things themselves.” (N_11)*

General practitioners, neurologists, MS nurses, nutritionists and sports scientists were named as the specialists who could be responsible for counseling on health behavior. A lack of time resources and missing evidence were identified as the biggest barriers to comprehensive lifestyle counseling. The difficulty of achieving long-term HBC in patients was also mentioned. Language barriers, the social environment and the workload of patients were cited as obstacles.

#### 3.1.2 Experienced health behavior changes by pwMS

**Figure 2:**
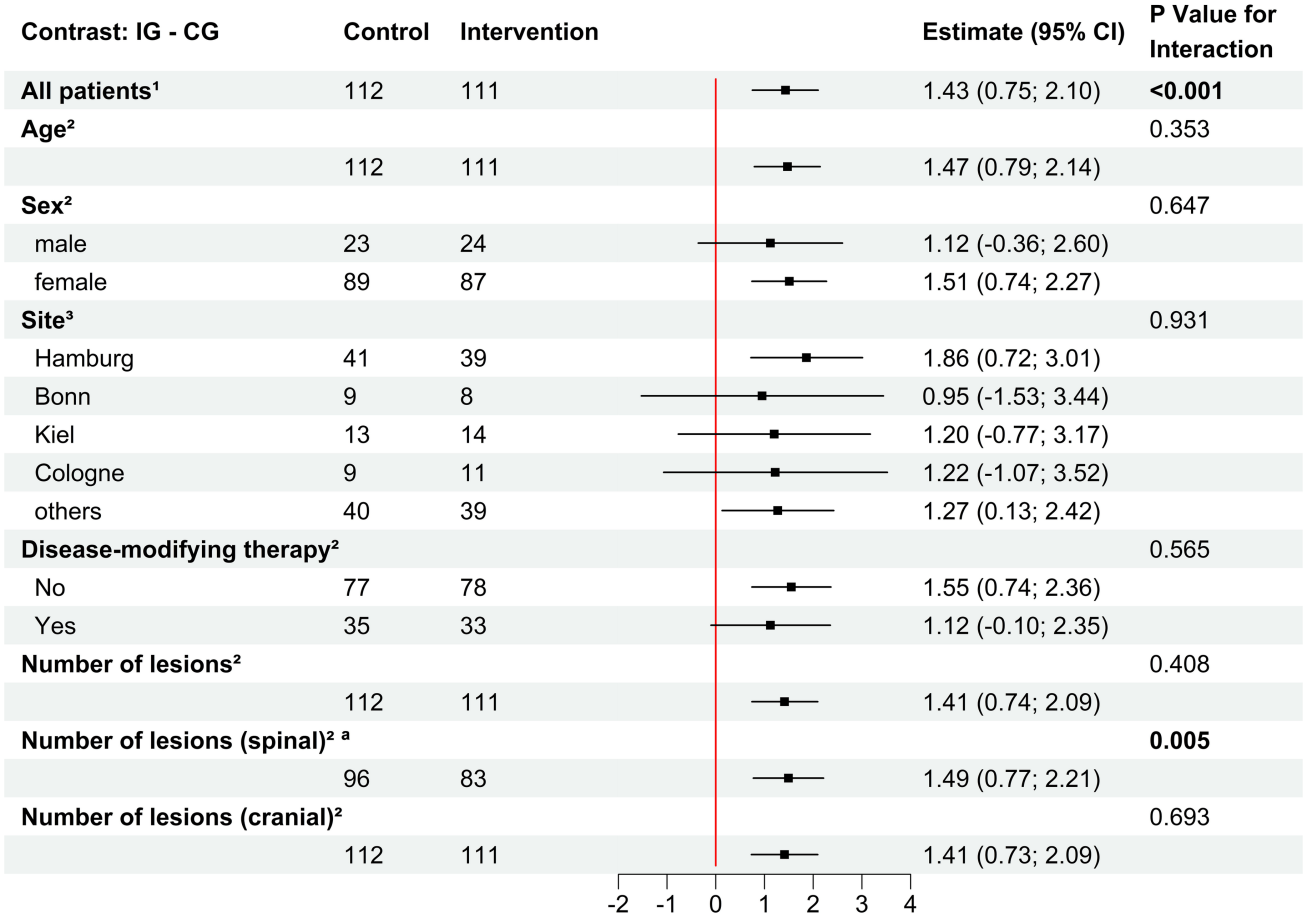
Effects of covariables (BL) on self-reported health behavior change (Month 3). Differences between intervention and control group were reported as pairwise contrasts of estimated marginal means derived from the linear model with 95% confidence interval and *p*-value for interaction and are visualized within a forest plot. CG, Control group; IG, Intervention group; CI, Confidence Interval. ^1^Month 3; Missing values, *n* = 11. ^2^Baseline; Missing values adjusted to independent variable. ^a^Missing value, *n* = 44.

Three months after randomization, pwMS were asked if they had already made changes in their health behavior on a scale from 0 to 10 (0 = do not agree and 10 = fully agree). PwMS using levidex reported a significantly higher agreement [IG (6.7); CG (5.2)]; least squares mean difference = 1.43 (95%-CI 0.75–2.10, *p* < 0.001). This is also reflected in all estimates in Figure 2, CI being >1. An ANOVA was conducted to examine the effect of the baseline data in terms of age, sex, center, lesion number and immunotherapy status on self-reported HBC, showing no relevant impact of these factors. The only factor showing between-group differences based on this analysis was the number of spinal lesions contributing to self-reported behavior change (*F* (1,175) = 8.25, *p* = 0.005). To further investigate the influence of spinal lesions, a Spearman correlation analyses with the Expanded Disability Status Scale, Hamburg Quality of Life Questionnaire Multiple Sclerosis and the Hospital Anxiety and Depression Scale were carried out (15–17). However, all correlation coefficients were either negligible or very weak (*ρ* < 0.15), which is why we assume the effect to be a chance finding. After 3 months, participants were asked about the specific areas in which they had already changed their behavior. 66.7% of pwMS in the IG reported that they already had a better ability to cope with stress (49.6% in the CG), 64% had changed their dietary behavior (53.1% in the CG), 53.2% changed their level of physical activity (50.4% in the CG) and 25.2% changed their sleeping patterns (14.2% in the CG). These descriptive differences in self-reported HBC could be reproduced at 6 months, but after 12 months, there were no differences (see Table 3).

**Table 3:** Patients PEQ self-reported behavior change.

Self-reported behavioral change of people with MS	Month 3 *N* = 224			Month 6 *N* = 223			Month 12 *N* = 218		
	levidex *n* = 111	dexilev *n* = 113	*p-*value	levidex *n* = 111	dexilev *n* = 112	*p-*value	levidex *n* = 107	dexilev *n* = 111	*p-*value
I have changed my health behavior since POWER@MS1.^1^	6.7 (2.4)	5.2 (2.8)	**<0.001**	6.7 (2.5)	6.1 (2.5)	0.08	n.e	n.e	-
I already changed my exercise behavior.^2^	53.2 (59)^2^	50.4 (57)^2^	0.85	59.5 (66)^2^	49.1 (55)^2^	0.37	7.4 (2.0)^1^	7.5 (2.0)^1^	0.54
I already changed my dietary behavior.^2^	64.0 (71)^2^	53.1 (60)^2^	0.34	70.3 (78)^2^	58.0 (65)^2^	0.32	7.0 (2.4)^1^	7.0 (2.7)^1^	0.99
I already changed my sleeping pattern.^2^	25.2 (28)^2^	14.2 (16)^2^	0.07	27 (30)^2^	24.1 (27)^2^	0.79	6.2 (2.2)^1^	6.0 (2.5)^1^	0.52
I already cope better with stress.^2^	66.7 (74)^2^	49.6 (56)^2^	0.12	65.8 (73)^2^	52.7 (59)^2^	0.26	6.1 (2.5)^1^	5.8 (2.4)^1^	0.37

n.e, not enquired; SD, Standard deviation. Items assessed on a Likert scale ranging from 0 to 10, group comparisons were conducted using independent-samples t-tests. For items with multiple categorical response options, group comparisons were performed using chi-square tests. Statistically significant *p*-values (*p* < 0.05) are highlighted in bold.

1 VAS rating on a scale from 0 to 10, where ‘0’ is no knowledge/disagree and ‘10’ is great knowledge/agree completely (mean/SD).

2 Question about changes in lifestyle in various areas (multiple choice possible) percentage of agreement in % (n).

Mean self-reported MS knowledge was 4.2 in the IG and 4.5 in the CG at baseline on a scale from 0 equals “no knowledge at all” and 10 equals “a lot of knowledge.” At months 3, 6, and 12, self-perceived MS knowledge was significantly higher in the dexilev group than in the levidex group (see Supplementary material E).

When interviewed, most pwMS stated that they believe be able to influence the disease progression through behavioral changes to some extent. Respondents from both groups stated lifestyle to be highly relevant to them. In particular, the areas of stress management and nutrition were perceived as important.

The following key strategies for HBC were identified in the levidex-group: Incorporating ‘cues’ in everyday life (e.g., reminders on the fridge), mindfulness and meditation exercises (audios) from levidex, guided exercise and goal setting. Concrete exercise plans and support from the partner were highlighted as important facilitators for change in physical activity in both groups.

*“But the fact that I could use the app and then just listen to and perform one of these relaxation techniques helped me a lot” (Power@MS1-101)*

Obstacles to lifestyle changes mentioned included MS symptoms (e.g., pain, fatigue), social in-fluence (e.g., participation in events, family life), the weather (heat, rain) and organizational as well as financial challenges in everyday life (e.g., stress, new structures, work).

### 3.2 Perception of digital health applications

#### 3.2.1 Health care professionals

In the questionnaires, 6 months after randomization up to 90% agreed or agreed somewhat that lifestyle counseling via an information platform can be helpful. One HCP reported that a pwMS criticized the text-heavy nature of and the lack of multimedia content during counseling within the DHA used in the study. Other HCPs criticized the extensive content and time efforts required for program completion.

When asked about their opinion on DHA as a supporting tool for HBC, they revealed ambivalent views. Most commonly, it was noted that DHA could not replace personal contact with treating physicians and eight out of 14 respondents emphasized the importance of the social component of counseling. Concerns were expressed that pwMS with impairments might have difficulties using DHAs. It was noted that personal contact would be an essential beneficial component that would be missing in a purely digital setting.

*“Nowadays, there's a lot of talk about more interaction, video, etc. I mean, it was really well done, no question, but it was also very text-heavy somewhere” (N_17)*

In general, participating HCPs indicated a positive and open attitude toward DHA. Advantages of DHA included the provision of neutral and structured information with a reliable level of evidence. Further, it was noted that DHA could enable continuous support without high personnel costs.

#### 3.2.2 People with MS

In the PEQs, pwMS were asked about their experiences with the information platforms, while the interview also covered their general acceptance of and experience with DHAs. After 12 months of usage, pwMS rated that the information platform was easy to navigate on a scale from 0 to 10 (0 = do not agree and 10 fully agree), with a mean of 7.7 in IG und 7.1 in the CG. Moreover, on the same scale, they rated that the provided information on the platform was easy to comprehend (8.5 in IG and 8.1 in CG). The total number of days with activity was 31 (42.1) days in the IG and 10.5 (4.1) days in CG (see Table 2). When asked if there were specific components that helped them to implement changes, 44 (39%) in the IG and 22 (30%) in the CG agreed. The mindfulness exercises (*n* = 9), stress management tips (*n* = 7), weekly text messages as well as the option of weekly self-monitoring questionnaires with a visible statistic over time (*n* = 5) were rated as particularly helpful in levidex. In dexilev, information on exercise and nutrition (*n* = 10) were rated as helpful.

When interviewed, levidex was rated as user-friendly. Attributes such as “intuitive to use” and “good design” were mentioned, and pwMS also stated that they felt that they were being taken seriously by the program. For dexilev, the constant availability was highlighted as a key advantage. However, criticism was raised regarding the heavy reliance on text. Additionally, some users found the content either already familiar or too superficial.

*“But when I think about how many hours I've spent on this online portal over the months, if a doctor would have had to tell me all this. Firstly, I would have been overwhelmed if it had all happened in one conversation. And secondly, who is supposed to do that?” (Power@MS1-132)*

The easily understandable description of complex issues, the continuous counseling, the monitoring of habits and the motivating design were positively emphasized. Two participants wished for a better approach for certain patient subgroups (i.e., parents). Further, pwMS wished for a chat within the intervention to enable direct enquiries.

*“Well, I was actually a bit worried that the portions would be too big to work on. But it was actually well measured. It was always like that, you could also interrupt in between if it was a bit much, because that's always a matter of concentration.” (Power@MS1-149)*

Levidex was also criticized for its heavy reliance on text. In addition, some of the content was already familiar to the users or was perceived as too superficial. The digital format was rated as appropriate for counseling, but the majority would have still preferred personal consultation. They indicated that levidex could be a good addition to the existing options of care but should not replace face-to-face consultations.

### 3.3 General feedback on POWER@MS1

#### 3.3.1 Health care professionals

In the PEQ, HCPs were asked about reasons for participation and the additional burden of the study in everyday practice. 40% stated that they were convinced of health behavior interventions and 7% stated that health behavior interventions would lower their communication burden with pwMS. Six months after randomization, nearly three-quarters of SN disagreed or somewhat disagreed that scheduling visits (79%) or MRIs (71%) was a stressor.

The interviews also revealed that the well-prepared study program and the good communication with the study center were particularly appreciated by HCPs. Overall, participation in the study was perceived as positive, even though HCPs did not indicate any relief in everyday working life due to the interventions. Different opinions were expressed on the benefits of the study in terms of advantages in care. Two neurologists noticed changes in the consultations with their patients and one stated that patients participating in the study asked more specific questions. Another neurologist commented that they saved time in terms of lifestyle education. In contrast, other HCPs could not recognize any changes during the clinical encounters or stated that the time saved on education was consumed by documentation efforts. Additionally, one neurologist reported that the collaboration with radiologists has intensified due to the study, another recommended a longer follow-up time. Moreover, it was criticized that the electronic Case Report Form (eCRF) used during the study had poor usability.

#### 3.3.2 People with MS

At all-time points of the process evaluation, more than 80% of pwMS stated that the study was not or rather not stressful for them. Twelve months after randomization, participants were asked to identify which study arm they believed they had been assigned to. In the IG, 41% of pwMS correctly identified levidex as the intervention program, while 59% mistakenly believed levidex represented the control program. In the CG, 56% of patients accurately assumed they had received the control program, while 44% incorrectly believed they had been using the intervention program.

During the interviews, pwMS stated that they perceived the study as well organized. Participants in both groups felt well looked after and described the study as rather calming or as a point of orientation after MS diagnosis. They did not perceive any impact of the study on consultations with their treating neurologists. Ten pwMS in the IG stated that levidex helped them to make HBCs and to gain a better understanding of the disease. Uncertainty regarding blinding was mentioned as a point of criticism. Also, the completion of questionnaires on paper was criticized - a digital solution was desired. SecuTrial® was commented as difficult to use, the different passwords used for the eCRF and intervention−/control program were also points mentioned to be improved.

### 3.4 Radiologists

Data from the interviews and the survey corresponded well. Participation in the study was not perceived as burdensome by 79%. All radiologists reported to use a specific MS examination standard in their practice, of which 85% classify according to Mc Donald 2017 and 68% check the spatial dissemination of lesions according to Swanton criteria.

During the interviews, radiologists rated close MRI checks alongside clinical visits as important and useful for monitoring of disease activity. They recommend carrying out MRIs more frequently than in an annual cycle (e.g., every 6 months) and primarily without the use of contrast agents to prevent pathological gadolinium deposits. Further, they rated a standardization of spinal MRI imaging as useful.

## 4 Discussion

This quantitative and qualitative process evaluation among pwMS and HCPs examined the perceived relevance and acceptance of digital health behavior interventions in MS in general as well as in relation to the POWER@MS1 study, in which the levidex and dexilev programs were used. A key result was that all participants considered health behaviors highly relevant in MS treatment. HCPs emphasized that the importance of HBC is not addressed sufficiently in current standard care. However, HCPs desired more reliable and precise evidence with regard to the impact of behavior change on disease progression in MS. Even though MS-specific guidelines for physical activity have been published by Kalb et al. (17), more precise recommendations would be appreciated. Similar conclusions were made in the review by Wang et al. (18), who identified a lack of specific guidelines as a barrier to recommendations in clinical practice. Another review by Marck et al. (19) recommends the use of action-oriented guidelines regarding physical activity, which may also be relevant for other lifestyle aspects such as nutrition, sleep and stress management. Apart from smoking, evidence for physical activity is relatively stronger than for other health behaviors. Nutrition and stress management were areas of key interest among pwMS participating in the POWER@MS1 study. In the process evaluation, pwMS reported substantial HBC after 3 and 6 month after randomization, whereas this was not reflected in most of the outcomes of the main study (10). However, pwMS in the IG reported significantly more self-perceived HBC than those in the CG, which was shown most clearly for a self-reported change of dietary and stress behavior. Since 59% of the participants in the IG and 44% in the CG believed they belonged to the other group, it can be concluded that blinding was largely effective, and that the observed effect is unlikely due to a lack of blinding. The discrepancy between the reported extent of HBC in the interviews and the questionnaire could be due to biased self-perception, social desirability on the patient’s side, or retrospective bias, as the interviews with pwMS were conducted up to 11 months after the end of the POWER@MS1 study. There is still a lack of responsive and objective outcome measures for health behaviors and the negative study findings might be due to the lack of sensitivity of the measures such as the used questionnaires (20). Given the heterogeneity and complexity of dietary habits, exercise patterns, or stress management, it seems conceivable that some participants made independent behavioral changes after engaging with levidex that were not well captured by the relatively broad and generic questionnaires employed in the POWER@MS1 study. In MS research, there are no agreed-upon objective measures for physical activity; subjective and often poorly validated self-reports as well as the conceptual diversity of these habits are major obstacles here (21), pointing to the need for further research.

Although in Germany DHA have been regulated rigorously since 2020, when the so-called “DiGA” directory was introduced that lists reimbursable programs that are deemed safe and effective, their implementation in routine care is still in its infancy (22). Especially integration into clinical communication processes poses considerable challenges (23). Feedback in POWER@MS1 demonstrates that HCPs and pwMS appreciate a digital tool as an adjunct to personal counseling in managing health behaviors. However, stand-alone digital support was considered not-sufficient. This was also observed in an earlier study of Daniel et al. providing digital support for increasing physical activity (24). HCPs and pwMS reported only a limited impact of digital content on medical encounters. Here, possibly more multimedia content within the digital tool might lead to better integration in care (25). While digitalization is a key priority in health management, particularly for chronic diseases, substantial evidence gaps remain. To date, only 13 randomized controlled trials of mobile health interventions for MS have been conducted—many of them pilot studies—with major limitations, including publication bias, inconsistent reporting, and a lack of long-term outcome data (26–28). The text-heavy format of levidex was criticized by some participants, while audio-recorded exercises were highly appreciated by most. The potential of video-based health information for people with chronic diseases was also considered in a review by Deshpande et al. (29). While the review found that video-based interventions can effectively improve short-term patient knowledge, it also highlighted that their impact on self-efficacy, HBC and long-term health outcomes is less consistent and often comparable to other forms of information, such as text or audios. This aligns with previous reviews that showed some promise but no clear superiority of video-based health behavioral interventions (30). In this context, future applications should carefully consider the strengths and limitations of different communication channels, recognizing the different preferences of the users, which might be interactive, text-based, audio or video formats. In general, evidence suggest that tailoring health behavioral interventions to individual needs and preferences tends to be more effective than offering generic interventions that are not custom-tailored (31, 32), suggesting that it might be sensible to offer video, text, or other channels specifically to those who prefer or can benefit from the respective channel. Nevertheless, previous research has demonstrated positive effects of levidex, including significant improvements in quality of life (33), suggesting that personalized digital interventions can play a valuable role in empowering pwMS to take an active role in their health.

Apart from suggesting opportunities for improving the digital intervention, this process evaluation also revealed other findings, such as that the trial design was considered acceptable, whereas the suitability of the digital outcome platform used was viewed as limited. The close monitoring by MRI and frequent neurological encounters were perceived as an advantage and may have promoted a sense of control by pwMS.

### 4.1 Limitations

A key limitation is that presumably HCPs as well as pwMS already had a higher level of interest in behavioral changes in the participating centers, which might not reflect the overall opinions and knowledge of MS-specialized neurologists. This is reflected in the high rate of neurologists considering health behaviors as equally relevant as immunotherapies. All pwMS recruited for the interviews had above-average user activity compared to the overall study population, which could have led to an overestimation of the relevance as well as ceiling effects, limiting the study’s power to detect improvements in health behavioral habits because the majority of participants already showed relatively healthy behaviors even at the start of the study. Due to the comparatively high baseline quality of life in early MS, detecting significant changes was challenging. Moreover, as levidex was compared to another psychoeducational intervention (dexilev), potential group differences may have been masked. Finally, this study only presents subjective data. An integration of subjective and objective outcomes in a comparative model might entangle interactions and effects of the interventions which were not yet detectable.

## 5 Conclusion

The process evaluation of the POWER@MS1 RCT shows that levidex, an MS-specific digital lifestyle intervention, was appreciated by HCPs and pwMS and seems promising in supporting HBC. There is an urgent need for the development of sensitive and valid outcome measures that can detect and quantify the health behavioral changes that some of the participants reported with greater precision. Overall, this study showed that digital health interventions like levidex hold promise for supporting meaningful health behavior changes in pwMS.

## Data Availability

The original contributions presented in the study are included in the article/Supplementary material, further inquiries can be directed to the corresponding author.

## Group members of the POWER@MS1 study group

Dr. Dieter Bähr (MS-Zentrum Klinikum Neukölln); Prof Dr. Achim Berthele, Veronika Grundner (Klinik für Neurologie, TUM Universitätsklinikum, München); Dr. Wendelin Blersch (Neurologische Praxis, Regensburg); Prof Dr. Achim Gass (UMM Universitätsmedizin Mannheim, MCTN Mannheim Center of Translational Neurosciences); Dr. Klaus Gehring (Neurozentrum am Klosterforst); PD Dr. Matthias Grothe (Universitätsmedizin Greifswald, Klinik und Poliklinik für Neurologie); Prof Dr. Kerstin Hellwig (Klinikum der Ruhr-Universität Bochum); Dr. Boris-Alexander Kallmann (Kallmann Neurologie); PD Dr. Juliane Klehmet (Jüdisches Krankenhaus Berlin); Dr. Schulamith Krüger (Helios Klinikum Uelzen); Prof Dr. Mathias Mäurer (Klinikum Würzburg-Mitte gGmbH); Dr. Stefan Meya (Nervenärztliche Gemeinschaftspraxis Alte/Hahne/Dr. Meya); Prof Dr. Patrick Oschmann (Klinikum Bayreuth GmbH, Klinik für Neurologie); Dr. Refik Pul (Universitätsklinikum Essen); Dr. Holger Roick (E/M/S/A – Zentrum f. Neurologie/Psychiatrie/Neuroradiologie); Prof Dr. Stephan Schmidt (Neurologische Gemeinschaftspraxis Bonn); Prof Dr. Andreas Steinbrecher (Helios Klinikum Erfurt GmbH, Klinik für Neurologie); PD Dr. Klarissa H. Stürner, Christian Ihlefeld (UKSH Campus Kiel, Klinik für Neurologie); Prof Dr. Clemens Warnke, Dr. Yasemin Göreci (Philipps University Marburg and Department of Neurology, University Hospital Gießen and Marburg).
