# Supplementary material for: Factors influencing health behavior changes in people with early multiple sclerosis: process evaluation of the multicenter randomized controlled POWER@MS1 trial
Source: Front Neurol. 2025 Oct 27;16:1635872. doi: 10.3389/fneur.2025.1635872 (PMC12599142; doi:10.3389/fneur.2025.1635872)
Supplement: Supplementary file 1 [file Data_Sheet_1.docx]

Supplementary Material

**Supplement A** Overview Process Evaluation POWER@MS1

**Supplement B**  Self-developed questionnaires: process evaluation

**Supplement C**  Semi-structured interview guide

**Supplement D** Category system of the telephone interviews for process evaluation POWER@MS1

**Supplement E** Evaluation of MS Knowledge, Usability, and Comprehension of Information Across Groups

**Supplement A:** Overview Process Evaluation POWER@MS1
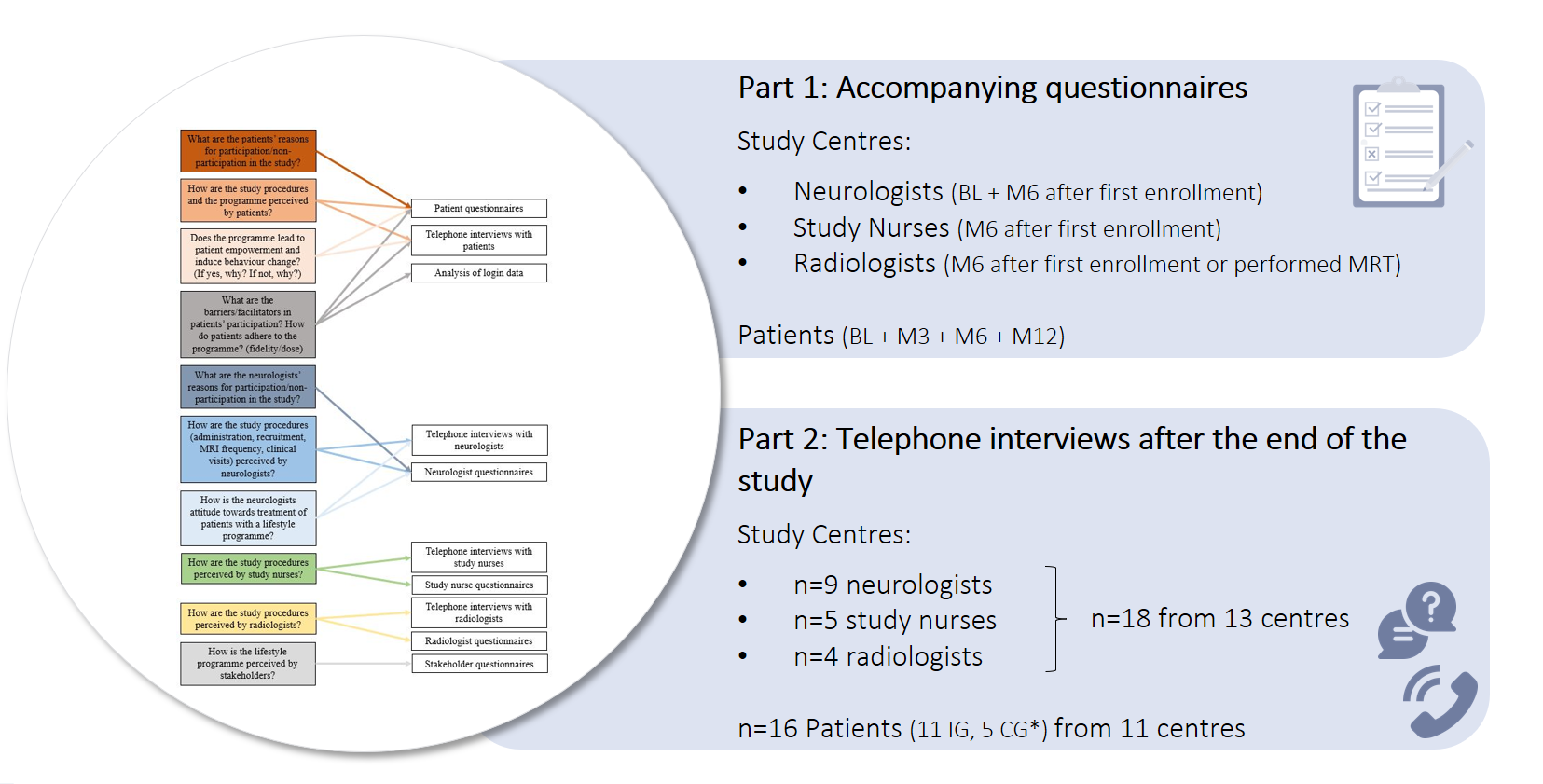


Figure 1 Process evaluation: Data collection times and groups (*IG = intervention group CG=control group)

**Supplement B: Self-developed questionnaires: process evaluation**

1. **Patients**
2. **Process evaluation (BL)**

Dear participant,

Thank you for taking the time to complete the questionnaire. Your answers will help us to obtain important information in order to better assess the results of the study. Please answer all questions.

**1. Why did you decide to participate in the study?** (multiple answers possible)

I think it's important that studies are carried out.

I would like to advance MS research with my participation.

I think the POWER@MS1 study is interesting.

☐ Other reasons:________________________________________

**2. How often do you use the Internet to find out about something?**

more than 5 times a week

2-5-times a week

maximum 1 time per week

maximum 1 time per month

Less than 1 time per month

never

**3. Have you already read up on multiple sclerosis?**

☐ No, I haven't read up on MS yet.

☐ Yes, via forums of those affected.

Yes, through the pharmaceutical industry.

Yes, via the German Multiple Sclerosis Society (DMSG)/ other patient organisations

☐ Yes, via other sources (please name) __________________________________

**4. Please rate your knowledge about multiple sclerosis.**

|  | **0** | **1** | **2** | **3** | **4** | **5** | **6** | **7** | **8** | **9** | **10** | a lot |
| --- | --- | --- | --- | --- | --- | --- | --- | --- | --- | --- | --- | --- |
| No knowledge at all |  |  |  |  |  |  |  |  |  |  |  | of knowledge |
|  |  |  |  |  |  |  |  |  |  |  |  |  |

The following statements refer to the goals that you associate with participation in the POWER@MS1 study. Please give an answer to each statement.

**5. I believe the information platform will help me to find my own way with the disease.**

| Do | **0** | **1** | **2** | **3** | **4** | **5** | **6** | **7** | **8** | **9** | **10** | Fully |
| --- | --- | --- | --- | --- | --- | --- | --- | --- | --- | --- | --- | --- |
| not agree at all |  |  |  |  |  |  |  |  |  |  |  | agree |

**6. I would like to take a closer look at the possibilities of immunotherapy for multiple sclerosis.**

| Do | **0** | **1** | **2** | **3** | **4** | **5** | **6** | **7** | **8** | **9** | **10** | Fully |
| --- | --- | --- | --- | --- | --- | --- | --- | --- | --- | --- | --- | --- |
| not agree at all |  |  |  |  |  |  |  |  |  |  |  | agree |

**7. I would like to take a closer look at the options for coping with my disease.**

|  | **0** | **1** | **2** | **3** | **4** | **5** | **6** | **7** | **8** | **9** | **10** | Fully |
| --- | --- | --- | --- | --- | --- | --- | --- | --- | --- | --- | --- | --- |
| Do not agree at all |  |  |  |  |  |  |  |  |  |  |  | agree |

**8. I would like to take a closer look at the possibilities of lifestyle changes for multiple sclerosis.**

|  | **0** | **1** | **2** | **3** | **4** | **5** | **6** | **7** | **8** | **9** | **10** | Fully |
| --- | --- | --- | --- | --- | --- | --- | --- | --- | --- | --- | --- | --- |
| Do not agree at all |  |  |  |  |  |  |  |  |  |  |  | agree |

**9. I would like to change my exercise behaviour.**

|  | **0** | **1** | **2** | **3** | **4** | **5** | **6** | **7** | **8** | **9** | **10** | Fully |
| --- | --- | --- | --- | --- | --- | --- | --- | --- | --- | --- | --- | --- |
| Do not agree at all |  |  |  |  |  |  |  |  |  |  |  | agree |

**10. I would like to change my nutritional behaviour.**

|  | **0** | **1** | **2** | **3** | **4** | **5** | **6** | **7** | **8** | **9** | **10** | Fully |
| --- | --- | --- | --- | --- | --- | --- | --- | --- | --- | --- | --- | --- |
| Do not agree at all |  |  |  |  |  |  |  |  |  |  |  | agree |

**11. I would like to change my sleeping patterns.**

|  | **0** | **1** | **2** | **3** | **4** | **5** | **6** | **7** | **8** | **9** | **10** | Fully |
| --- | --- | --- | --- | --- | --- | --- | --- | --- | --- | --- | --- | --- |
| Do not agree at all |  |  |  |  |  |  |  |  |  |  |  | agree |

**12. I would like to learn how to deal with stress better.**

|  | **0** | **1** | **2** | **3** | **4** | **5** | **6** | **7** | **8** | **9** | **10** | Fully |
| --- | --- | --- | --- | --- | --- | --- | --- | --- | --- | --- | --- | --- |
| Do not agree at all |  |  |  |  |  |  |  |  |  |  |  | agree |

1. **Process evaluation (month 3)**

Dear participant,

Thank you for taking the time to complete the questionnaire. Your answers will help us to obtain important information in order to better assess the results of the study. Please answer all questions. **The questions refer to the last three months.**

1. **How often have you used the information platform in the last three months?**

More than ten times

Five tot en times

Once to four times

Never

**1a. I found my way around the information platform easily.**

| Do | **0** | **1** | **2** | **3** | **4** | **5** | **6** | **7** | **8** | **9** | **10** | Fully |
| --- | --- | --- | --- | --- | --- | --- | --- | --- | --- | --- | --- | --- |
| not agree at all |  |  |  |  |  |  |  |  |  |  |  | agree |

**1b. I was able to understand the information on the platform.**

| Do | **0** | **1** | **2** | **3** | **4** | **5** | **6** | **7** | **8** | **9** | **10** | Fully |
| --- | --- | --- | --- | --- | --- | --- | --- | --- | --- | --- | --- | --- |
| not agree at all |  |  |  |  |  |  |  |  |  |  |  | agree |

**1c. Please rate your knowledge about multiple sclerosis.**

| No | **0** | **1** | **2** | **3** | **4** | **5** | **6** | **7** | **8** | **9** | **10** | a lot |
| --- | --- | --- | --- | --- | --- | --- | --- | --- | --- | --- | --- | --- |
| knowledge at all |  |  |  |  |  |  |  |  |  |  |  | of knowledge |

1. **The number of visits to the doctor as a result of the study is stressful for me.**

☐ Agree

☐ Tent to agree

☐ Tent to disagree

☐ Disagree

1. **The number of MRIs through the study is stressful for me.**

☐ Agree

☐ Tent to agree

☐ Tent to disagree

☐ Disagree

1. **Editing the content on the information platform is stressful for me.**

☐ Agree

☐ Tent to agree

☐ Tent to disagree

☐ Disagree

1. **The number of questionnaires I have to fill in is a burden for me.**

☐ Agree

☐ Tent to agree

☐ Tent to disagree

☐ Disagree

1. **Der the overall effort involved in the POWER@MS1 study (visits to the doctor, MRIs, processing the content of the information platform and completing the questionnaires) is stressful for me.**

☐ Agree

☐ Tent to agree

☐ Tent to disagree

☐ Disagree

1. **Have you informed yourself about additional treatment options for MS in the last six months?** (multiple answers possible)

☐ No, I did not inform myself about MS therapies.

☐ Yes, via forums of those affected.

Yes, via pharmaindustry.

Yes, via the German Multiple Sclerosis Society (DMSG)/ other patient organisations

☐ Yes via other sources___________________________________

1. **Have you obtained additional information about lifestyle changes for MS in the last three months?** (multiple answers possible)

☐ No, I haven't done any additional research.

☐ Ja, via forums of those affected.

Yes, via pharma industry.

Yes, via the German Multiple Sclerosis Society (DMSG)/ other patient organisations

☐ Yes via other sources___________________________________

1. **Since taking part in the POWER@MS1 study, I have already made some changes to my lifestyle.**

| Do | **0** | **1** | **2** | **3** | **4** | **5** | **6** | **7** | **8** | **9** | **10** | Fully |
| --- | --- | --- | --- | --- | --- | --- | --- | --- | --- | --- | --- | --- |
| not agree at all |  |  |  |  |  |  |  |  |  |  |  | agree |

Please only answer question 11 if you have already made changes to your lifestyle.

1. **In which areas have you already made changes to your lifestyle since taking part in the POWER@MS1 study?** (multiple answers possible)

☐ Coping with the disease

☐ Physical activity behaviour

☐ Dietary behaviour

☐ Sleeping patterns

☐ Other: __________________________________

Thank you very much for your help!

1. **Process evaluation (Month 12.1)**

Dear participant,

Thank you for taking the time to complete the questionnaire. Your answers will help us to obtain important information in order to better assess the results of the study. Please answer all questions.

**The questions refer to the last six months.**

1. **How often have you used the information platform in the last six months?**

More than ten times

Five tot en times

Once to four times

Never

**1a. I found my way around the information platform easily.**

| Do | **0** | **1** | **2** | **3** | **4** | **5** | **6** | **7** | **8** | **9** | **10** | Fully |
| --- | --- | --- | --- | --- | --- | --- | --- | --- | --- | --- | --- | --- |
| not agree at all |  |  |  |  |  |  |  |  |  |  |  | agree |

**1b. I understood the information on the platform very well.**

| Do | **0** | **1** | **2** | **3** | **4** | **5** | **6** | **7** | **8** | **9** | **10** | Fully |
| --- | --- | --- | --- | --- | --- | --- | --- | --- | --- | --- | --- | --- |
| not agree at all |  |  |  |  |  |  |  |  |  |  |  | agree |

1. **The emails and text messages I received through the information platform were helpful to me.**

| Do | **0** | **1** | **2** | **3** | **4** | **5** | **6** | **7** | **8** | **9** | **10** | Fully |
| --- | --- | --- | --- | --- | --- | --- | --- | --- | --- | --- | --- | --- |
| not agree at all |  |  |  |  |  |  |  |  |  |  |  | agree |

1. **Please rate your knowledge about multiple sclerosis.**

| No | | **0** | | **1** | | **2** | | **3** | | **4** | **5** | **6** | **7** | | **8** | | **9** | | **10** | | a lot | |
| --- | --- | --- | --- | --- | --- | --- | --- | --- | --- | --- | --- | --- | --- | --- | --- | --- | --- | --- | --- | --- | --- | --- |
| knowledge at all | |  | |  | |  | |  | |  |  |  |  | |  | |  | |  | | of knowledge | |
|  |  | |  | |  | |  | |  | |  |  | |  | |  | |  | |  | |  |

1. **The number of visits to the doctor as a result of the study is stressful for m**

☐ Agree

☐ Tent to agree

☐ Tent to disagree

☐ Disagree

1. **The number of MRIs through the study is stressful for me.**

☐ Agree

☐ Tent to agree

☐ Tent to disagree

☐ Disagree

1. **Editing the content on the information platform is stressful for me**

☐ Agree

☐ Tent to agree

☐ Tent to disagree

☐ Disagree

1. **The number of questionnaires I have to fill in is a burden for me**

☐ Agree

☐ Tent to agree

☐ Tent to disagree

☐ Disagree

1. **The overall effort involved in the POWER@MS1 study (visits to the doctor, MRIs, processing the content of the information platform and completing the questionnaires) is stressful for me.**

☐ Agree

☐ Tent to agree

☐ Tent to disagree

☐ Disagree

1. **Have you informed yourself about additional treatment options for MS in the last six months?**

☐ No, I did not inform myself about MS therapies.

☐ Yes, via forums of those affected.

Yes, via pharmaindustry.

Yes, via the German Multiple Sclerosis Society (DMSG)/ other patient organisations

☐ Yes via other sources___________________________________

1. **Have you obtained additional information about lifestyle changes for MS in the last three months?** (multiple answers possible)

☐ No, I haven't done any additional research.

☐ Yes, via forums of those affected.

Yes, via pharmaindustry.

Yes, via the German Multiple Sclerosis Society (DMSG)/ other patient organisations

☐ Yes, via other sources___________________________________

1. **The study compared two different information platforms on health behaviour in MS:** **An information platform with standard information and a newly developed information platform.** **Which group do you think you were in?**

intervention group - i.e. new information platform

control group – i.e Information platform with standard information

Thank you very much for your help!

1. **Patient: process evaluation (Month 12.2)**

Dear participant,

Thank you for taking the time to complete the questionnaire. Your answers will help us to obtain important information in order to better assess the results of the study. Please answer all questions.

1. **I have set myself goals to change my health behaviour.**

☐ Agree

☐ Tent to agree

☐ Tent to disagree

☐ Disagree

1. **I have already made concrete plans to implement a change in my health behaviour.**

☐ Agree

☐ Tent to agree

☐ Tent to disagree

☐ Disagree

1. **I have changed my health behaviour (e.g. diet, exercise or sleeping patterns).**

☐ Agree

☐ Tent to agree

☐ Tent to disagree

☐ Disagree

**Please only answer questions 4 to 10 if you have made changes to your health behaviour.** **Otherwise, please continue with question 11**

1. **I can cope better with the illness.**

| Do | **0** | **1** | **2** | **3** | **4** | **5** | **6** | **7** | **8** | **9** | **10** | Fully |
| --- | --- | --- | --- | --- | --- | --- | --- | --- | --- | --- | --- | --- |
| not agree at all |  |  |  |  |  |  |  |  |  |  |  | agree |

1. **I have changed my exercise behaviour.**

| Do | **0** | **1** | **2** | **3** | **4** | **5** | **6** | **7** | **8** | **9** | **10** | Fully |
| --- | --- | --- | --- | --- | --- | --- | --- | --- | --- | --- | --- | --- |
| not agree at all |  |  |  |  |  |  |  |  |  |  |  | agree |

1. **I can cope better with stress.**

| Do | **0** | **1** | **2** | **3** | **4** | **5** | **6** | **7** | **8** | **9** | **10** | Fully |
| --- | --- | --- | --- | --- | --- | --- | --- | --- | --- | --- | --- | --- |
| not agree at all |  |  |  |  |  |  |  |  |  |  |  | agree |

1. **I have changed my dietary behaviour.**

| Do | **0** | **1** | **2** | **3** | **4** | **5** | **6** | **7** | **8** | **9** | **10** | Fully |
| --- | --- | --- | --- | --- | --- | --- | --- | --- | --- | --- | --- | --- |
| not agree at all |  |  |  |  |  |  |  |  |  |  |  | agree |

1. **I have changed my sleeping patterns.**

| Do | **0** | **1** | **2** | **3** | **4** | **5** | **6** | **7** | **8** | **9** | **10** | Fully |
| --- | --- | --- | --- | --- | --- | --- | --- | --- | --- | --- | --- | --- |
| not agree at all |  |  |  |  |  |  |  |  |  |  |  | agree |

1. **I have resolved to maintain the changes in my lifestyle permanently**

☐ Agree

☐ Tent to agree

☐ Tent to disagree

☐ Disagree

1. **Was there a specific component on the platform that particularly helped you to implement the change?**

☐ Yes

☐ No

**If yes, please explain***:*

_______________________________________________________________________________________________________________________________________________________________________________________________________________________________________________________________

1. **What has made it difficult for you to change your health behaviour?** (Please explain briefly)

__________________________________________________________________________________________________________________________________________________________________________________________________________________________________________________________________

1. **What would have made it easier for you to change your lifestyle?** (Please explain briefly)

________________________________________________________________________________________________________________________________________________________________________________________________________________________________________________________________

1. **HCP’s**
2. **neurologist (Baseline)**


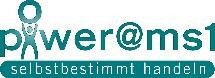


Process evaluation

(To be completed, once for BL, by each doctor at the centre)

Dear participant,

Thank you for taking the time to complete the questionnaire. Your answers will help us to obtain important information in order to better assess the results of the study. Completing the questionnaire should not take more than 10 minutes of your time. Please answer all questions.

1. **What is your sex?**

☐ female

☐ male

☐ divers

1. **How old are you?** __________years

1. **How long have you been working in the outpatient clinic/practice?**

_________ (month and year)

1. **Please state your function in the outpatient clinic/practice**:

☐ Neurologists

☐ Senior doctor

☐ Intern

☐ Other (please name) _______________________

1. **What is your MS knowlege?**

☐ excellent (expert)

☐ very extensive

☐ moderate

☐ limited/low

1. **Please estimate how many MS patients you see each week:**

_____ patients

1. **Please estimate how many MS patients you talk to each week about lifestyle changes (e.g. exercise, diet):**

_____ patients

1. **Health behaviour factors contribute to the disease activity of MS.**

☐ Agree

☐ Tent to agree

☐ Tent to disagree

☐ Disagree

1. **The course of MS is influenced by health behaviour factors.**

☐ Agree

☐ Tent to agree

☐ Tent to disagree

☐ Disagree

1. **If a patient is well informed, I can accept their decision, even if I would recommend something else.**

☐ Agree

☐ Tent to agree

☐ Tent to disagree

☐ Disagree

1. **In principle, all relapsing-remitting MS patients should start immunotherapy.**

☐ Agree

☐ Tent to agree

☐ Tent to disagree

☐ Disagree

1. **Please briefly describe the circumstances in which you would be in favour of a patient not wanting to start immunotherapy initially:**

__________________________________________________________________________

__________________________________________________________________________ __________________________________________________________________________

__________________________________________________________________________

1. **Why are you participating in the study? (Multiple answers possible)**

☐ I think the study is intresting

☐ I am convinced of health behavior change

☐ I believe that lifestyle intervention will take the pressure off me.

☐ Other: _____________________________________________________

_____________________________________________________

_____________________________________________________

Thank you for your co-operation!Please file this document in study folder 1 under process evaluation and document the form in the eCRF (via secuTrial®).

1. **neurologists (month 6)**

Dear participant,

Thank you for taking the time to complete the questionnaire. Your answers will help us to obtain important information in order to better assess the results of the study. Completing the questionnaire should not take more than 10 minutes of your time. Please answer all questions.

1. **What is your MS knowlege?**

☐ excellent (expert)

☐ very extensive

☐ moderate

☐ limited/low

1. **Please estimate how many MS patients you discuss health behavior change options (e.g. exercise, diet) with each week.:** _____ patients

1. **Please estimate how many patients you have included in the study so far:**

____patients

1. **Does your workflow change on POWER@MS1-recruitment days?**

☐ Yes

☐ No

☐ Somewhat

If **yes** or **somewhat** please disvcribe:

_____________________________________________________________________

_____________________________________________________________________

_____________________________________________________________________

1. **It is very difficult to interrupt the conversation with patients to inform them about the study.**

☐ Agree

☐ Tent to agree

☐ Tent to disagree

☐ Disagree

1. **The additional organisational work involved in the study is a burden for me.**

☐ Agree

☐ Tent to agree

☐ Tent to disagree

☐ Disagree

Please describe the load in more detail if necessary:

__________________________________________________________________________

__________________________________________________________________________

1. **The number of clinical visits due to the study is stressful for me.**

☐ Agree

☐ Tent to agree

☐ Tent to disagree

☐ Disagree

1. **The number of MRI’s due to the study is stressful for me.**

☐ Agree

☐ Tent to agree

☐Tent to disagree

☐ Disagree

1. **I am relieved when a patient takes part in POWER@MS1.**

☐ Agree

☐ Tent to agree

☐Tent to disagree

☐ Disagree

1. **I think it's good that the study is being carried out in our outpatient clinic/practice**

☐ Agree

☐ Tent to agree

☐ Tent to disagree

☐ Disagree

Please describe your position in more detail:

_______________________________________________________________________________________________________________________________________________

1. **Health behaviour counselling via an information platform is helpful for patients.**

☐ Agree

☐ Tent to agree

☐ Tent to disagree

☐ Disagree

Please describe your position in more detail:

_____________________________________________________________________

_____________________________________________________________________

1. **The information platform enables patients to participate more actively in decisions about immunotherapies.**

☐ Agree

☐ Tent to agree

☐ Don’t know

☐ Tent to disagree

☐ Disagree

Thank you for your assistance

Please file this document in study folder 1 under process evaluation and document the form in the eCRF (via ^secuTrial®^).

1. **
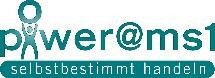
study nurse (month 6)**

**Process evaluation study nurse**

(To be completed, once for V3, by each participating study nurse at the centre)

Dear participant,

Thank you for taking the time to complete the questionnaire. Your answers will help us to obtain important information in order to better assess the results of the study. Completing the questionnaire should not take more than 10 minutes of your time. Please answer all questions**.**

1. **What is your sex?**

☐ female

☐ male

☐ divers

1. **How old are you?** __________years

1. **How long have you been working in the outpatient clinic/practice?**

_________ (month and year)

1. **What is your MS knowlege?**

☐ Excellent (expert)

☐ Very extensive

☐ Moderate

☐ Limited/low

1. **Does your workflow change on POWER@MS1-recruitment days?**

☐ Yes

☐ No

☐ Somewhat

If **yes** or **somewhat** please describe:

_____________________________________________________________________

_____________________________________________________________________

1. **I was approached more frequently by patients about health behavior as a result of the study.**

☐ Agree

☐ Tent to agree

☐ Tent to disagree

☐ Disagree

1. **The additional organisational work involved in the study is a burden for me.**

☐ Agree

☐ Tent to agree

☐ Tent to disagree

☐ Disagree

Please describe the load in more detail if necessary:

__________________________________________________________________________

__________________________________________________________________________

1. **The number of clinical visits due to the study is stressful for me.**

☐ Agree

☐ Tent to agree

☐ Tent to disagree

☐ Disagree

1. **The number of MRI’s due to the study is stressful for me.**

☐ Agree

☐ Tent to agree

☐Tent to disagree

☐ Disagree

Please describe the burden brifly:

_____________________________________________________________________

_____________________________________________________________________

1. **I think it's good that the study is being carried out in our outpatient clinic/practice**

☐ Agree

☐ Tent to agree

☐Tent to disagree

☐ Disagree

Please describe your position in more detail:

__________________________________________________________________________

__________________________________________________________________________

1. **Beratungen zum Lebensstil durch eine Informationsplattform sind hilfreich für die Patienten.**

☐ Agree

☐ Tent to agree

☐Tent to disagree

☐ Disagree

Please describe your position in more detail:

________________________________________________________________________

________________________________________________________________________

Thank you for your assistance.

Please file this document in study folder 1 under process evaluation and document the form in the eCRF (via ^secuTrial®^).

1. **radiologists**

**Process evaluation radiology**

(To be completed, once for V3, by each participating radiologist)

Dear participant,

Thank you for taking the time to complete the questionnaire. Your answers will help us to obtain important information in order to better assess the results of the study. Completing the questionnaire should not take more than 10 minutes of your time. Please answer all questions**.**

1. **What is your sex?**

☐ female

☐ male

☐ divers

1. **How old are you?** __________years

1. **How long have you been working in radiology?** ___ ______ (month and year)

1. **Please indicate your function in the radiological practice an:**

☐ radiologist

☐ Other (Please name) _______________________

1. **What is your MS knowlege?**

☐ Excellent (expert)

☐ Very extensive

☐ Moderate

☐ Limited/low

1. **The additional organisational work involved in the study is a burden for me.**

☐ Agree

☐ Tent to agree

☐ Tent to disagree

☐ Disagree

Please describe the load in more detail if necessary:

_____________________________________________________________________

_____________________________________________________________________

1. **I think it is important to have close-interval MRI examinations in the early stages of the disease.**

☐ Agree

☐ Tent to agree

☐ Tent to disagree

☐ Disagree

1. **I think it's good that the study is being carried out in collaboration with our radiological practice.**

☐ Agree

☐ Tent to agree

☐ Tent to disagree

☐ Disagree

Please describe your position in more details:

__________________________________________________________________________

__________________________________________________________________________

1. **Is there a special MS examination standard in your radiological practice?**

☐ Yes

☐ No

1. **Do you classify MS according to McDonald 2017 criteria?**

☐ Yes

☐ No

1. **Do you count lesions??**

☐ Yes

☐ No

1. **Check the spatial dissemination of lesions according to Swanton criteria?**

☐ Yes

☐ No

Thank you for your assistance

**Supplement C: Semi-structured interview guide**

| **Approximate Time** | 30 minutes |
| --- | --- |
| **Information and consent** | Signed before the start of the interview |
| **Introduction, organisational questions, basics 10 min** | |
| General introduction & procedure: | ‘Thank you very much for taking the time for this interview. Your answers will help us to gain important information in order to better categorise the results of the POWER@MS1 study.’  - The duration of the interview is approximately 30 minutes  - The interview will be recorded  - All data will be treated confidentially  - When the data is analysed later, all information is anonymised so that no identification is possible  - Questions?  Do you agree with this?  Get feedback! |
| **Start of recording** | |
| Introduction to the topic | ‘Our aim today is to reflect on the implementation of the POWERMS1 study and to record your impressions. You can talk to me openly, because this is the only way we can learn and improve.’ |

1. **neurologists**

| **Part I –**  **Background & questions on participation in POWER@MS1** | | |
| --- | --- | --- |
| Leading question (narrative prompt) | Check - Was this mentioned? Possible enquiry if not addressed by itself | Maintenance and control issues |
| **Please tell me why you took part in POWER@MS1?**  **What tasks did you take on as a neurologist as part of the implementation of the POWER@MS1 study?**  **How did you perceive the preparation for the implementation of the POWER@MS1 study? (e.g. initiation, documents...)** | -Was it your own decision to take part in the study as a centre or were you asked to participate?  -What were your expectations when you took part?  e.g. recruitment, information, organisation of the visits...?  -If not well prepared:  Looking back, is there anything you wish you had known or wished you had known before participating in POWER@MS1?  What would have helped you to feel better prepared?Haben Ihnen Informationen gefehlt? | Can you explain this in a little more detail? |

| **Part II – POWER@MS1:**  **Study administration (recruitment & study planning/implementation)** | | |
| --- | --- | --- |
| Leading question (narrative prompt) | Check - Was this mentioned? Possible enquiry if not addressed by itself | Maintenance and control issues |
| **How did the inclusion of patients proceed at your centre?**  **To what extent has conducting the POWER@MS1 study affected your everyday work?** | -How do you rate the interest in the study?  -Were there any difficulties with recruitment?  -If recruitment was difficult:  What were the reasons for this?  What problems did you encounter?  Could these be solved? If so, how?  Were there any unresolved problems?  How did you perceive the organisational effort involved in POWER@MS1?  e.g. planning and implementation of MRIs or clinical rounds  -For people who perceived the additional organisational effort as a burden (Check ProEva questionnaire):  What were the reasons for this?  In your opinion, how could the additional workload be reduced? | -And then?  -Can you go into a little more detail here? |

| **Part III –**  **Relevance of & barriers to lifestyle counselling** | | |
| --- | --- | --- |
| Leading question (narrative prompt) | Check - Was this mentioned? Possible enquiry if not addressed by itself | Maintenance and control issues |
| **How do you rate the relevance of patient counselling on lifestyle and MS in general?**  **How often do your patients ask questions about the influence of lifestyle on MS?**  **To what extent do you see lifestyle counselling for MS patients as part of your medical duties?**  **What lifestyle services are available at your centre? (in addition to POWER@MS1)**  **Are there any barriers that make it difficult for you to advise patients on lifestyle and MS?**  **What are your thoughts on the concept of ‘lifestyle optimisation instead of early immunotherapy’ in the first 1-2 years after an MS diagnosis?** | -With high perceived relevance:  What clinical goals do you think can be achieved by optimising lifestyle? (patient autonomy, self-efficacy, quality of life)  -Are there certain areas about which questions are asked particularly frequently, e.g. sport, nutrition...?  -Do you feel responsible for counselling patients in these areas?  -Do you see a particular speciality as having a special responsibility?  -Do your patients have access to lifestyle counselling, information material...?  -With specific offers: Are there ‘best practice’ examples that have particularly helped your patients to optimise their lifestyle?  What has proved particularly successful?  -Are there barriers such as time pressure/lack of income for lifestyle counselling?  If so, which ones?  And how do you think these barriers could be overcome?  -Do you follow this concept in your centre?  -What do you see as the advantages/disadvantages? | Can you explain this in more detail? |

| **Part IV – POWER@MS1:**  **Influence of the study/optimisation potential & attitude towards digital lifestyle interventions** | | |
| --- | --- | --- |
| Leading question (narrative prompt) | Check - Was this mentioned? Possible enquiry if not addressed by itself | Maintenance and control issues |
| **Are there any aspects that have been improved by POWER@MS1 in your centre and have led to a reduction in workload?**  **(Check ProEva questionnaire)**  **Looking back, do you think it was good that your centre took part in POWER@MS1?**  **What could possibly be improved?**  **Has your attitude towards digital lifestyle interventions for people with MS changed as a result of the POWER@MS1 study?**  **Is there perhaps another counselling concept that you think would be better suited to providing lifestyle information and achieving behaviour change than a DiGA?**  **The intervention programme ‘levidex’ is now provisionally available as a prescribable DiGA: Would you recommend levidex to your patients in the future and prescribe it via a prescription?** | -If so, how? (For example, in your experience, did the participants feel better cared for or supported and had less need for counselling?)  -If not, what were the reasons for this? (For example, did the participants ask more questions about lifestyle and MS, resulting in additional work?)  (Check ProEva questionnaire)  If yes/no, why?  -If yes, in what way?  -What do you see as the advantages of a DiGA?  -What are the limitations of a DiGA?  If yes, which one? (e.g. personal/online counselling)  -People who find lifestyle counselling through a digital intervention rather unhelpful for patients (Check ProEva questionnaire):  What are the reasons for this?  -If yes/no, why?  -If no, what would it take? | Can you explain this in more detail? |

| **Part V –**  **Additions / Conclusion (neurologists/study nurses and radiologists)** | | |
| --- | --- | --- |
| Leading question (narrative prompt) | Check - Was this mentioned? Possible enquiry if not addressed by itself | Maintenance and control issues |
| **Are there any other aspects that have not yet been addressed and that you would like to add?** |  |  |

1. **study nurses**

| **Part I –**  **Relevance of & barriers to lifestyle counselling** | | |
| --- | --- | --- |
| Leading question (narrative prompt) | Check - Was this mentioned? Possible enquiry if not addressed by itself | Maintenance and control issues |
| **How do you assess the relevance of patient counselling on lifestyle and MS in general?**  **How high is the demand for lifestyle counselling by patients in your centre?**  **Does the topic of lifestyle occur in your centre?**  **Are there ‘best practice’ examples that have helped your patients to optimise their lifestyle?**  **If so, which ones?**  **What barriers do you recognise in your day-to-day work that make it difficult for you to advise patients on lifestyle and MS?**  **And how do you think these barriers could be overcome?** | -With high perceived relevance:  What clinical goals do you think can be achieved by optimising lifestyle? (patient autonomy, self-efficacy, quality of life...)  -How often do your patients ask questions of their own accord about lifestyle options?  -Do they feel responsible for counselling patients?  -What lifestyle services are available at your centre? (in addition to POWER@MS1)  -Do your patients have access to lifestyle counselling, information material...?  (only for centres where lifestyle is present)  -Are there barriers such as time pressure/lack of resources for lifestyle counselling? | Can you explain this in more detail? |

| **Part II – POWER@MS1:**  **Activities & preparation** | | |
| --- | --- | --- |
| Leading question (narrative prompt) | Check - Was this mentioned? Possible enquiry if not addressed by itself | Maintenance and control issues |
| **What tasks did you take on as a study nurse during the implementation of the POWER@MS1 study?**  **Did you feel sufficiently informed about the POWER@MS1 study and prepared for its implementation?**  **Looking back, is there anything you wish you had known or wished you had known before participating in POWER@MS1?** | e.g. recruitment, planning of MRIs/visits...?  -If no, what would have helped you to feel better informed/prepared?  -If yes, what? | Can you explain this in a little more detail? |

| **Part III – POWER@MS1: Perception of study administration (recruitment & study planning/implementation)** | | |
| --- | --- | --- |
| Leading question (narrative prompt) | Check - Was this mentioned? Possible enquiry if not addressed by itself | Maintenance and control issues |
| **How did you perceive the recruitment of patients for the POWER@MS1 study?**  **Did the implementation of the POWER@MS1 study influence your usual working day?**  **How did you interact with the medical management on this topic?**  **How did you perceive the organisational effort involved in POWER@MS1?** | -How do you estimate the interest in the study?  -With difficult recruitment:  What were the reasons for this?  What problems did you encounter?  Could these be solved? If so, how?  Were there any unresolved problems?  If so, how?  e.g. planning of MRIs or clinical rounds, documentation of rounds & patient questionnaires  -For people who found the additional organisational workload/number of visits/number of MRIs stressful (Check ProEva questionnaire):  What were the reasons for this?  In your opinion, how could the additional workload be reduced? | -And then?  -Can you go into a little more detail here? |

| **Part IV – POWER@MS1: Influence of the study, optimisation potential & attitude towards digital lifestyle interventions** | | |
| --- | --- | --- |
| Leading question (narrative prompt) | Check - Was this mentioned? Possible enquiry if not addressed by itself | Maintenance and control issues |
| **Looking back, do you think it was good that your centre took part in POWER@MS1?**  **Looking back, to what extent would you want to do something differently/what could possibly be improved?**  **What was your opinion on digital lifestyle interventions for people with MS before the POWER@MS1 study?**  **Has your attitude changed as a result of the study?**  **Can you think of a tool that might be better suited to providing lifestyle information and achieving behavioural change than a DiGA?**  **The intervention programme ‘levidex’ is now provisionally available as a medical product that can be prescribed: Would you recommend levidex to patients in the future?** | *(Check ProEva questionnaire)*  *-If yes/no, what are the reasons for this?*  *-What do you see as the advantages of a DiGA?*  *-Do you have any concerns about using a DiGA? If yes, which ones?*  *-Study nurses who find lifestyle counselling via a DiGA rather unhelpful for patients (Check ProEva questionnaire):*  *What are the reasons for this?*  *-If yes, which one?*  *-If yes/no, why?*  *-If no, what would it take?* | Can you explain this in more detail? |

1. **radiologists**

| **Part I –**  **Background & questions on participation in POWER@MS1** | | |
| --- | --- | --- |
| Leading question (narrative prompt) | Check - Was this mentioned? Possible enquiry if not addressed by itself | Maintenance and control issues |
| **Why did you participate in POWER@MS1 as a radiologist?**  **Were you informed about the contents of the study by the centre?**  **How did you perceive the preparation for the implementation of the POWER@MS1 study? (e.g. initiation, documents...)**  **How do you assess the relevance of close-meshed MRI examinations in the early stages of MS (Check ProEva questionnaire)?** | -Ask for initial thoughts on the project in order to obtain a picture of the mood (negative/ indifferent/ positive):  What did you think?  -Voluntary participation:  What were your motives?  - What information do you know about the POWER@MS1 study? (e.g. content and objectives of the study)  -Looking back, is there anything you wish you had known before participating in POWER@MS1?  -What would have helped you to feel better prepared?  -Did you miss any information?  -Do you think people with MS should have more frequent MRI scans within the first 1-2 years?  -In your opinion, are there any reasons that speak against close MRI examinations? | Can you explain this in more detail? |

| **Part II – Perception of MRI** | | |
| --- | --- | --- |
| Leading question (narrative prompt) | Check - Was this mentioned? Possible enquiry if not addressed by itself | Maintenance and control issues |
| **What did a typical POWER@MS1 study MRI appointment look like?**  **How did you perceive the organisational effort involved in the POWER@MS1 study?**  **Has a study MRI had an impact on the team's usual work process?**  **Do you classify in MS according to McDonald 2017?**  **Do you check the spatial dissemination of lesions in MS according to Swanton criteria?** | -Have you had any contact with participants yourself?  -Were there enough free slots available?  -Was it easy to send the CDs? (clear name/pseudonym)  -If additional organisational work was perceived as burdensome (Check ProEva questionnaire):  What are the reasons for this?  How could the effort have been reduced?  If yes, how?  If yes, why do you think this makes sense?  If not, for what reasons?  If no, how do you categorise instead?  If yes, why do you think this makes sense?  If not, for what reasons?  If no, how do you check the spatial dissemination instead? | -And then?  -Can you go into a little more detail here? |

| **Part III –**  **MRI standard and conclusion on POWER@MS1 & study participation** | | |
| --- | --- | --- |
| Leading question (narrative prompt) | Check - Was this mentioned? Possible enquiry if not addressed by itself | Maintenance and control issues |
| **How appropriate did you find the MRI examinations in the course of the POWER@MS1 study?**  **As with cranial MRIs, could you also imagine a nationally applicable spinal MRI standard for MS?**  **Do you think that radiology in general can and should be involved in conducting studies?**  **Looking back, how do you feel about being involved in POWER@MS1?**  **What advice would you give us for planning a similar study with regard to the involvement of radiology?** | -Was the frequency of MRIs appropriate (a total of 5 MRIs over 2 years: 4 in the 1st year & 2 in the 2nd year)?  -Was the specified MRI standard appropriate or rather difficult to implement in the care setting? (particularly relevant for radiology with a different MRI standard)  -Was the financing appropriate?  Background: spinal imaging was an idea in the course of the POWER@MS1 study, which was discarded due to the current lack of standards and the resulting difficulties in implementation.  (currently only sagittal images + transversal if required)  -What is your opinion specifically on the involvement of radiology in neurological/MS studies?  -Is there anything that could possibly be improved? | -Können Sie das etwas genauer erklären? |

1. **patients**

| **Planned** duration | 20 - 30 minutes |
| --- | --- |
| **Information and consent** | Signed before the start of the interview |
| **Introduction, organisational questions, basics 10 min** | |
| General introduction & procedure: | "Thank you very much for taking the time for this interview. Your answers will help us to gain important information in order to better categorise the results of the POWER@MS1 study."   - The duration of the interview is approximately 30-45 minutes - The interview will be recorded - All data is treated confidentially - When the data is subsequently analysed, all details are anonymised so that no identification is possible - Questions?   Do you agree with this?  *Get feedback!*  Start recording |
| **Start of recording** | |
| Introduction to the topic | "Our aim today is to reflect on the implementation of the POWERMS1 study and to record your impressions. In particular, we are also interested in your impression of the online programme as part of healthcare. You can talk to me openly, because this is the only way we can learn something and improve it for future studies." |

Interview guide - [patients]

| **Part Ia - Reasons for participation, organisational aspects & interaction** | | |
| --- | --- | --- |
| Leading question (narrative prompt) | Check - Was this mentioned? Possible enquiry if not addressed by itself | Maintenance and control issues |
| **How did you hear about the POWER@MS1 study?**  **How did you experience the POWER@MS1 study?**  **Did you feel sufficiently informed and prepared to participate in the study?**  **Please tell me how you perceived the care you received during the study.**  **What role did the POWER@MS1 study play in the time after your MS diagnosis?** | - What aroused your interest in the study?  - How did you feel about the study?  - Looking back, is there anything that you wish you had known or wished you had known before you took part?  - Is there anything you would have imagined differently?  - Was the study staff easily accessible?  - Were the attending physicians easily accessible?  - Where would you have liked more support?  - To what extent did participating in the study influence the time after your diagnosis?  - Has participation changed your understanding of your MS disease and/or the way you deal with it?  - If no influence, what would have helped you in the time after your diagnosis? | Can you explain this in more detail?  ***Check Eval. Month 12***  Was the programme (IG/KG) recognised? No □ Yes □ Yes  e.g. support in coping with illness  Study dropout? |

| **Part Ib - Reasons for participation, organisational aspects & interaction** | | |
| --- | --- | --- |
| Leading question (narrative prompt) | Check - Was this mentioned? Possible enquiry if not addressed by itself | Maintenance and control issues |
| **What motivated you personally to stay involved until the end of the study?**  **Have your conversations with your neurologist changed as a result of participating in the study?** | - To what extent did the online programme (levidex/dexilev) play a role in this?  - To what extent did the care provided by more frequent visits and MRIs play a role in this?  - What did you find more important during your time after your diagnosis? The online programme or the support?  - Did you enquire more or less about certain areas (e.g. lifestyle)?  - If so, do you have any examples?  - Were you able to ask more specific questions in the doctor's interview as a result of the study?  *- Did you have the impression that the nature of the dialogue had changed and that you were more actively involved in decisions, for example?* | Can you explain this in more detail?  e.g. support in coping with illness  Study dropout? |

| **Part II - POWER@MS1: Lifestyle and behaviour change** | | | |
| --- | --- | --- | --- |
| Leading question (narrative prompt) | Check - Was this mentioned? Possible enquiry if not addressed by itself | Maintenance and control issues | |
| **What role does lifestyle play for you, e.g. exercise, diet or sleep, in relation to your management of MS?**  **Did you change anything in your lifestyle behaviour during the study?**  (e.g. more exercise/sport, healthier diet, better handling of stress, better sleep hygiene**)**  **Do you currently still need lifestyle advice?** | - Why do you think lifestyle is important/not so important when dealing with MS?  - Which lifestyle area is most important to you?  - Has the study changed your assessment of how important the topic is?  If yes:  - What exactly have you changed?  - How did you realise this?  - What obstacles did you encounter in the process?  - What in particular helped you to implement these changes?  - Have you been able to maintain the changes in the long term?  - If not, what would have helped you to maintain the changes in the long term?  If no:  - For what reasons have you been unable/unwilling to change your lifestyle?  - What obstacles did you encounter?  - Looking back, what would have helped you to overcome these obstacles?  - Looking back, what do you wish you had done to change your lifestyle?  *- If so,* in which areas?  - In what form would you like to be advised? | Can you please explain this in more detail?  ***Check Eval. & Outcomes Final Results***  Were there any change(s) in lifestyle behaviour based on the completed questionnaires?  □ No  □ Yes, namely (note for interview):  __________________________________  __________________________________  __________________________________  __________________________________  __________________________________  __________________________________ | |
|  |  |  | |
| **Part III - POWER@MS1: levidex (intervention group)** | | | |
| Leading question (narrative prompt) | Check - Was this mentioned? Possible enquiry if not addressed by itself | | Maintenance and control issues |
| **Please tell me about your personal impressions of levidex.**  **How well did you feel personally addressed by the levidex content?**  **You did not/rarely/often use levidex, is that correct? Can you please tell me the reasons why you did not/rarely/often use levidex and did/do not quit?**  **Have you used the additional materials provided in levidex?**  e.g. recipes, meditation instructions, sports exercises  **Have you used the optional SMS and/or e-mail messages that can be sent by levidex?**  **Do you think levidex is a suitable offer for first-time MS sufferers?** | - What expectations did you have of the programme and to what extent were these fulfilled or not fulfilled?  - What did you particularly like about it?  - What didn't you like about it?  - How helpful did you find levidex?  - How did you perceive the dialogue format?  - How did you feel about the fact that you could only use the calls once?  - How well could levidex be integrated into your everyday life?  - Looking back, is there anything you missed?  - Was there an area where you would have liked more information? (Psychological well-being, sleep, nutrition, exercise)  - What specific improvements or features would you like to see in future versions?  *If so,* which ones and how often?  *If not,* why did you not use them?  *- If so,* what did you think of this news?  *- If not,* for what reasons?  - Would you recommend levidex to other sufferers?  *- If so*, for what reasons?  *- If not,* what would it take for you to recommend levidex? | | - Can you please go into a little more detail here?  ***Check User Activity***  □ Not used at all  □ Rarely used, ___ Calls ended  □ Often used, ___ Calls ended  *- Would you recommend levidex even without personal contact (e.g. through more frequent visits)?* |

#

| **Part III - POWER@MS1: dexilev (control group)** | | |
| --- | --- | --- |
| Leading question (narrative prompt) | Check - Was this mentioned? Possible enquiry if not addressed by itself | Maintenance and control issues |
| **Please tell me about your personal impressions of dexilev.**  **How well did you feel personally addressed by the dexilev content?**  **You have ended/not ended dexilev, is that correct**?  **Can you please tell me about the reasons why you did/did not quit dexilev?** | - What did you particularly like about it?  - What didn't you like about it?  - How helpful did you find dexilev?  - How well could dexilev be integrated into your daily routine?  - Looking back, is there anything you missed?  - What would you want to change about dexilev?  - How did you feel about the fact that you could only use the modules once? | - Can you please go into a little more detail here?  ***Check User Activity***  □ Not used at all  □ Rarely used, ___ Calls ended  □ All calls ended, ___ Logins |

| **Part IV - POWER@MS1: Perception of DiGAs as an extension of the utility sector** | | |
| --- | --- | --- |
| Leading question (narrative prompt) | Check - Was this mentioned? Possible enquiry if not addressed by itself | Maintenance and control issues |
| **What experience did you have with digital programmes before the study?**  e.g. DiGAs, health apps, regular use of videos with sports instructions  **How did you feel about receiving lifestyle change support via a digital programme?**  **Has your attitude towards DiGAs changed as a result of participating in the POWER@MS1 study?**  **Under what circumstances would you favour a digital health application over more traditional forms of healthcare?** | - Has the coronavirus pandemic had an impact on your attitude to and use of DiGAs?  - Was the digital form of counselling enough for you?  - Is there perhaps a format that you think would be more suitable than a digital programme?  -If yes/no, to what extent has it changed?  -Where do you see the advantages of DiGAs?  -What do you see as the disadvantages of DiGAs?  - What role does personalised lifestyle advice from medical staff play for you?  - Is personalised advice important to you?  - If so, in which areas?  How often? (weekly, monthly...)  Who would you like to hear from personally  be counselled? (Doctors, nurses, peers...)  - For which areas related to your MS disease would information about digital programmes be sufficient for you? | - Can you please explain this in more detail?  *KG only: Would you be interested in getting access to the levidex programme? (Offer access as a thank you for participation? Or refer our patients to the possibility of being prescribed it!)* |

| **Part V - Supplements / Conclusion** | | |
| --- | --- | --- |
| Leading question (narrative prompt) | Check - Was this mentioned? Possible enquiry if not addressed by itself | Maintenance and control issues |
| **Looking back, how would you personally rate your participation in the POWER@MS1 study?**  **What suggestions for improvement would you give us?**  **Are there any other aspects that we have not yet discussed and that you would like to add in conclusion?** | -If good/not good, what are the reasons for this?  - Looking back, is there anything we could have done differently? |  |
| **Final information:** If you have any questions or further comments, please do not hesitate to contact me by phone/email. | | |

**Supplement D: Category system of the telephone interviews for process evaluation POWER@MS1**

1. **Patients (intervention group: n=11; control group: n=5)**

| **Categories & subcategories** | **Summary** | **Anchor examples** |
| --- | --- | --- |
| **General Feedback**   - Reasons for participation - Perception of participation - Perception of preparation - Perception of care - Role of the study after diagnosis - Reasons for motivation - Influence on the discussions with treating neurologists | In this category, patients were asked about their perception of the organisational processes, reasons for participation, support and the relevance of the study in the phase following MS diagnosis.  The patients surveyed cited the fact that the study represented an alternative to immunotherapy, the desire for scientifically sound information on the disease and regular monitoring as key factors in favour of participating in the study. Participation was predominantly rated positively by the respondents. The preparation was perceived as sufficient and the support as good to very good. The constant availability and rapid feedback on concerns were particularly positively emphasised. It was noted critically that in some centres the questionnaires could not be completed online. In addition, the documentation tool (secuTrial®) was found to be less user-friendly in some cases. Furthermore, the participants stated that the study gave them reassurance after the diagnosis, expanded their knowledge about the disease and encouraged them to make positive changes to their lifestyle to influence the course of the disease. A process of intensive engagement with the disease through the study was also described.  The participants in the intervention group cited the safety provided by the close MRI examinations and clinical visits, levidex itself (content, audio materials, weekly monitoring) and the connection to the study centres as reasons for staying in the study. In the control group, the close supervision and the willingness to support MS research were also cited as motivational factors.  Most participants reported that the study had no influence on their conversations with their treating neurologists. Some of the patients felt better prepared for the discussions or empowered as equal dialogue partners. | *‘That's why it was very important for me and ultimately also helpful in deciding on the medication that I'm now taking. I didn't take any back then and didn't want to take any. And now I've been thinking about it long enough to be able to decide.’ (Power@MS1-109)*  *‘But the POWER@MS study did me good because it was so much settled information, so filtered information, that it wasn't a lot of text, but it was just so to the point and that was good. Simply for the mind, I would say. (Power@MS1-208)*  *“I have to admit the app itself. The fact that there were always the parts where you could and had to do this check-in and then you could look at the graphs with the lines showing how your health behaviour had developed, how you felt, how your sleeping patterns were or whatever. A good trigger for me. It's like a computer game. Collecting points, so to speak.“ (Power@MS1-101)* |
| **Health behavior changes**   - The role of lifestyle in managing MS - Changes in lifestyle behaviour - Strategies/assistance for change - Barriers/hurdles to change - Current need for counselling | In this category, barriers and facilitators of intervention with regard to lifestyle change were addressed. The relevance of the topic for the patients as well as strategies and obstacles in the change process were discussed.  The patients (with one exception) stated that lifestyle was an area in which they felt they could influence the progression of the disease themselves. Respondents from both groups therefore categorised lifestyle as highly relevant. In particular, the areas of stress management and nutrition were mentioned several times.  With regard to changes made to lifestyle behaviour, the intervention group mentioned changes in dietary behaviour, stress management and more exercise in everyday life. Specifically, shopping and sleeping habits were changed, working hours were reduced and relaxation exercises were integrated into everyday life. In the control group, the measures mentioned were reducing sugar or meat consumption, taking dietary supplements and continuously increasing physical activity through online workouts.  The following strategies for changing lifestyle behaviour were identified in the intervention group: Incorporating ‘cues’ in everyday life (e.g. reminders on the fridge) to remind them to change their behaviour, mindfulness and meditation exercises (audios) from levidex, group exercise and SMART goal setting. Concrete exercise plans and support from the partner were mentioned in both groups. One person in each group also reported that the fear of disease progression acted as a motivator for implementing a healthier lifestyle.  Obstacles to lifestyle changes mentioned included symptoms of MS (e.g. pain, Uhthoff phenomenon), social influences (e.g. participation in events, family life), the weather (heat, rain) and organisational and financial challenges in everyday life (e.g. stress, new structures, work).  There were contradictory opinions in both groups regarding the need for further lifestyle counselling. While around half had no need for further counselling, the other half would have liked either regular counselling (on new research) or in-depth counselling on stress or nutrition. | *‘So the change in behaviour really came about through this tool, I would say. So maybe I could have done it on my own, because I think I can manage things like that reasonably well. But with this tool, it was definitely more possible and quicker.’ (Power@MS1-146)*  *‘But the fact that I was able to use the app and then simply listen to one of these relaxation techniques and do them helped me a lot. [...] I have developed the awareness to push myself less, but to pay more attention to organising myself in such a way that I have as little stress as possible, that I eat as healthily as possible, exercise regularly and do all this without stressing myself out.’ (Power@MS1-101)*  *‘And as long as these are not yet automatisms and habits, it is of course very easy to fall back into them. And that's why I thought it was really good that it was presented over such a relatively long period of time’ (Power@MS1-132)*  *‘‘When I think about it a bit, I would quite like to get advice on [nutrition and supplements] if there was someone who does that and what. But I think I know the basics now.’ (Power@MS1-146)* |
| **Feedback on levidex (intervention group)**   - Personal impressions - Integration into everyday life - Additional materials - Optional SMS & e-mails - Potential for improvement | The levidex intervention programme was described as intuitive to use and lovingly designed. The users felt that they were taken seriously and cared for by the insights gained from the programme. The simple description of complex issues, the continuous counselling, the monitoring of habits and the motivating design were particularly positively emphasised. In general, levidex was also described as easy to integrate into everyday life. The lack of availability as an app on smartphones and pre-download options for the content were criticised. The interview format was predominantly described as appealing, although some participants pointed out that the limited answer options were not always appropriate. The one-time availability was rated favourably by the participants, although the majority would have liked to have had longer access to the content. Audio exercises, weekly check-ins and worksheets were rated as helpful. The reminder emails and text messages sent by the programme were not used by the majority of participants. When they were used, they were found to be helpful. The dosage of content was perceived as good. There was criticism of the case studies of fictitious people integrated into the programme, which one person did not like. It was also reported that levidex did not focus enough on the lives of parents (especially mothers). The lack of an option to ask questions directly in a chat in levidex was also criticised. | *‚I thought it was really nice that the app introduced you to small steps and suggestions on how to slowly integrate new routines into everyday life. So I thought that was good or more in the app, it was well done ‘ (Power@MS1-179)*  *‘Well, I was actually a bit worried that the portions would be too big to work with. But it was actually well dosed. It was always like that, you could also interrupt in between if it was a bit much, because that's always a matter of concentration’ (Power@MS1-149)*  *‚Yes, if I remember correctly, it was just that I naturally represent a marginalised group here, which wasn't really well represented in the responses. For one thing, I'm a man. Statistically speaking, I think this disease is more common in women. On the other hand, I will soon be 50 or am approaching 50. ‘(Power@MS1-132)*  *‘Yes, I would have liked to do it again, because then it might have solidified even more’ (Power@MS1-109)* |
| **Feedback on dexilev (control group)**   - Personal impressions - Integration into everyday life - Potential for improvement - Personal approach | Overall, the user interface of the dexilev control group programme was described as deficient. Points of criticism included a heavy reliance on text. In addition, some of the content was already familiar to the users or was perceived as too superficial. The use of the programme could be flexibly integrated into everyday life. Participants expressed the wish to be able to access the content of the modules again afterwards. A better dosage of information within the modules, the desire for practical tips for implementation (e.g. recipes) and a reminder function were identified as possibilities for improvement. | “*Because there was so much text that I lost interest at some point.”(Power@MS1-075)*  *‘that you can experiment a bit in this app or on this site and then look okay, if I want to do sports now, then there are somehow websites where I can somehow YouTube, links or something, where I can do sports or if I look at nutrition, okay, here. Nutrition plan.“ (Power@MS1-208)* |
| **DHA as an extension**  **of the healthcare sector**   - Impact of COVID-19 - Previous experience with digital programmes - Perception of the digital format - Influence of the study on attitudes towards DHAs - Role of personal counselling - Digital vs. personal counselling | With regard to the COVID-19 pandemic as a contextual factor, it was shown that the patients participating in the interviews were still sceptical about digital applications before the pandemic or had no previous contact with DiGAs. Overall, they stated that they had developed a higher level of acceptance for digital applications and perceived them as a valuable offer in times of social isolation. In addition, the pandemic would have had the advantage that more time was available for use. One person reported a sharp decrease in mobility due to isolation.  The flexible use of DiGAs in terms of time and location and the associated ability to save on travelling were cited as advantages of using DiGAs. In addition, the amount of information provided, the ability to control it at one's own pace and the anonymity (e.g. less shame) when using it were mentioned. At the same time, anonymity was criticised by one person. Further points of criticism were the lack of individuality, the lack of opportunity to clarify questions and misunderstandings and the lack of pressure to be socially desirable (e.g. if the programme is used infrequently).  It was found that patients rated the digital format as appropriate for counselling, but that the majority of patients would still prefer a personal consultation to an app. The app would be a good addition to the range of care, but should not replace face-to-face consultations. | *‚I've only been at home the whole time and sat and somehow sat there cramped up, whatever. And this has actually caused the MS to deteriorate in terms of mobility ‘(Power@MS1-075)*  *‚* *Sometimes you just want to read through things in peace and quiet and take something out of it for yourself. Well, I thought it was quite nice to be able to do that in the evening, cosily on the sofa, so to speak, and think about it myself before talking to someone about it. ‘ (Power@MS1-233)*  *‚* *But when I think about how many hours I've spent on this online portal over the months, if a doctor had had to tell me all this. Firstly, I would have been overwhelmed if it had all happened in one conversation. And secondly, who is supposed to do that? ‘ (Power@MS1-132)*  *„So I think these digital apps are always an addition to everyday life, to the routine. But I think it's always necessary to have at least one check-in or at the beginning and at the end and in between a possibility to talk to a doctor or a counsellor or something like that“ (POWER@MS1-101)* |
| **Conclusion**   - Further information channels - Suggestions for improvement - Retrospective evaluation of participation - Final additions | The patients predominantly rated their participation in the POWER@MS1 study as positive. One suggestion for improvement was to enable digital responses to the study questionnaires. In addition, the desire for feedback on nutrition questionnaires was expressed. In retrospect, one person also pointed out that they had felt under pressure to optimise their health behaviour in various lifestyle areas. It was also noted that it would have been helpful if treating neurologists had been aware of the contents of the programme. | *„[…] it would just be good to have the option of asking questions directly online, for example, at the moment they come to you“ (Power@MS1-132)*  *„I thought that was very good and that it really helped me to consolidate everything and get into action and keep it that way in the long term. [...] Yes, exactly. I had the impression that I had put myself under a lot of pressure that I had to do everything right. “ (Power@MS1-146)* |

1. **neuologists (n=9) and study nurses (n=5)**

| **Categories & subcategories** | **Summary** | **Anchor examples** |
| --- | --- | --- |
| **Background and participation**   - Reasons for participation/expectations of participation - Tasks/responsibilities - Perception of the preparation for the realisation of the study | The neurologists gave several reasons for their participation, including interest in the concept, the scientifically sound patient education and the opportunity to save consultation time. In addition, the promotion of patient self-efficacy and the offer of a treatment option for patients who do not want immunotherapy were also mentioned.  In terms of task distribution, the study nurses mainly took on organisational tasks, such as scheduling and documenting visits and MRIs. The neurologists were responsible in particular for recruitment, information, documentation and conducting the visits.  The preparation of the study was predominantly rated positively by the centres surveyed. Points of criticism concerned the online documentation tool secuTrail®. The reason for this was poor usability. In addition, the wish was expressed for a clearer schedule at the beginning of the study (for initiation) and version numbers on all study documents. | *„Yes, by giving patients access to a neutral platform, so to speak, we can also provide patient education, so I think it really makes a lot of sense and of course saves us practitioners a lot of time overall.“ (N_08)*  *„And it is long overdue that there are alternatives to this [...] better information about their disease and that it is neutral, but also that it is made clear to patients from the outset that they are not helpless, but that there are massive possibilities, especially in the area of lifestyle factors“ (N_11)*  *„ [...]Then I was always told read this too [...] Yes, I don't even know how to explain it, it's not really to the point. So I have a question and I just want an answer. In other words, yes, it was a bit complex at the beginning“ (SN_08)* |
| **Perception of study administration**   - Recruitment - Influence on the usual daily work routine - Interaction with medical management (only surveyed for study nurses) | The patients who met the inclusion criteria were informed about the study by the neurologists during the ward rounds and given comprehensive information. In particular, the study was presented to patients who were sceptical about immunotherapies. Several neurologists pointed out a possible selection bias in this regard. In one centre, pre-screening was carried out for the presenting patients, whereby the study nurses placed information material in the files, for example, or contacted suitable patients by telephone after the visits.  The interest of patients in initial participation in the study was predominantly rated as very high by the centres. The following points were identified as key challenges in recruitment: the requirement that patients should be treatment-naive at the start, the impact of the COVID-19 pandemic and the lack of human and time resources.  Overall, the POWER@MS1 study did not have a major impact on the neurologists' day-to-day work and the additional workload was described as acceptable. This was also the case when no time savings (e.g. due to additional study documentation) were identified during consultations. The assessment of the study nurses varied greatly in the centres surveyed. For some study nurses, the study did not represent any additional effort, while others found the documentation effort and the organisation of the MRI appointments to be time-consuming. In order to reduce the documentation effort, the wish was expressed several times to carry out the documentation completely online. At the same time, one study nurse interviewed would have preferred purely paper-based documentation. The study nurses unanimously stated that the division of tasks and communication between neurologists and study nurses was clearly regulated. | *„Yes, we have now advertised in particular at the beginning for patients who may also be sceptical about immunotherapy, at the very beginning of the disease“ (N_12)*  *„[…]On Fridays, we do the pre-screening for the following week and then select the patients who are eligible for a study and then attach the relevant flyers to the files for the doctors to inform and educate the patients if necessary.“ (SN_04)*  *„And once that was done, we actually informed all newly diagnosed patients about it. And the number of those who took part was well over 90%. The willingness was very, very high.“(N_08)*  *„When you include a patient in a study like this, you become the patient's primary carer and in this respect it changes our everyday university life. And I would say that, but not in an unpleasant way.“ (N_12)*  *„So it was insane at the beginning. Simply because this, this jumble of paper that came so uncontrollably, it really kept me busy for hours. It really paralysed me at work. „(SN_13)* |
| **Relevance of and barriers to health behavior counselling**   - Relevance and clinical goals - Patient interest and areas of interest - Responsible specialities - Barriers to lifestyle counselling - Thoughts on the concept of lifestyle counselling vs. early immunotherapy - Existing lifestyle counselling | When asked about the clinical relevance of lifestyle factors for disease progression, it became clear that the respondents see these as a relevant factor in MS treatment. In particular, the positive effect of moderate exercise and sporting activity as well as the negative consequences of smoking were mentioned here. However, some respondents pointed to the current lack of scientific evidence on the effectiveness of specific lifestyle interventions, which makes it difficult to make specific recommendations.  The concept of lifestyle change with close monitoring instead of early immunotherapy was rejected by the majority of neurologists. Three interviewees stated that waiting could be justifiable in the case of a mild course of MS. Overall, lifestyle modification was seen more as an additive treatment measure.    Patient interest in MS-related lifestyle information was rated as very high by both neurologists and study nurses, especially in the first year after MS diagnosis. They reported that they were most frequently asked about nutrition. It was also noted that many patients read up on lifestyle aspects and were confused by the information available.  GPs, neurologists, MS nurses, nutritionists and sports scientists were named as the specialists responsible for lifestyle counselling.  A lack of time resources and a lack of evidence were identified as the biggest barriers to comprehensive lifestyle counselling. The difficulty of achieving long-term lifestyle changes in patients was also mentioned. Language barriers, the social environment and the workload of patients were cited as obstacles.  Existing lifestyle offers at the centres surveyed included group training, MS days with lectures, smoking cessation programmes (external), nutritional advice, sports studies and an MS mentoring programme. | *‘[...] lifestyle factors are extremely important, both objectively and psychologically, because they naturally give a little autonomy back to those affected’ (N_08)*  *“[…] but also that it is made clear to patients from the outset that they are not helpless, but that there are massive possibilities, especially in the area of lifestyle factors. To influence things themselves.” (N_11)*  *‘Well, I do think that ultimately, by optimising lifestyles and lifestyle factors, disease activity and also those that might reduce disease activity.’ (N_22)*  *„And there, we certainly address nutrition and exercise, but in a very general way and perhaps less specifically. However, if you are completely honest, there is still a lack of evidence for a specific intervention in many areas“ (N_12)*  *“I'm a bit skeptical, but let's say it's at least not 100% impossible that it might even have a causal effect.” (N_17))*  *“But now just from my. My personal experience is that MS patients are actually very well read. Are well read and know their way around. But many also read too much and have too many opinions, so that there is a bit of uncertainty.” (SN_08)*  *„I would actually not support that, that an adjustment of lifestyle factors is an adequate alternative to immunotherapy.”(N_08)*  *„If there are reasons not to start immunotherapy immediately, then information about lifestyle measures should definitely be provided and the patients should ultimately be taken along.“ (N_10)*  *„* *So my concept is basically always therapy, i.e. therapy AND lifestyle advice, but not either/or.“ (N_17)* |
| **Influence of the study & optimization potential**   - Improvement/relief through POWER@MS1 - Retrospective feedback on participation - Optimisation potential - Reasons for adherence | The respondents gave very diverse answers regarding the influence of the POWER@MS1 study on their day-to-day work. At one centre, cooperation with radiology was intensified. Furthermore, patients were given a sense of security on site. It was reported that consultation times with patients were shortened or more specific questions were asked by patients. However, three neurologists denied any time savings or reduced consultation times.  Overall, participation in the study was perceived as predominantly positive. The cooperation with the UKE was rated as pleasant and, according to the interviewees, the participating patients benefited from the study. The amendment was viewed critically by one person, but praised by another. Furthermore, a longer follow-up was requested.  There was potential for optimisation with regard to blinding, as the study arm was clearly recognisable for one person. It was also pointed out that several study accounts (study and documentation programme) were confusing for the participants. One access for everything would have been desirable.  The high adherence of the patients was attributed by the interviewees to the high need for counselling and the close support provided by clinical visits and MRIs.  The interviews revealed ambivalent opinions on digital health applications (DiGAs). The most common comment was that DiGAs could not replace personal contact with treating physicians. Concerns were expressed that patients with visual, sensory or motor impairments might have difficulties using the applications. It was also criticised that an essential part of treatment could be lost due to the lack of personal contact with carers. There was also concern that mental symptoms could not be recognised and treated in time due to the lack of personal contact, as technical applications cannot fully take over diagnostics in this area.  The advantages of DiGAs were cited as being able to provide neutral and structured information with a reliable level of evidence. The constant availability in everyday life and the associated accessibility were also emphasised. It was also mentioned that DiGAs could enable continuous support without high personnel costs due to their everyday use.  The attitude towards DiGAs has changed among the interviewees to the extent that one interviewee only became aware of the existence of these services as a result of the study. For the most part, the participants indicated a positive and open attitude towards DiGAs. Two people stated that they were still sceptical, as there was still no scientific evidence of the effectiveness of levidex. However, a large proportion of respondents continued to value personal counselling as an important factor and considered DiGAs to be merely a useful addition to the treatment spectrum.  Seven of the respondents stated that they would prescribe or recommend levidex. This was based on the positive feedback from the patients participating in the study, the desire to educate MS sufferers and to provide evidence-based information. One person stated that they would only prescribe levidex on request. The reason given was that the evidence situation was still unclear at the time of the interview. | *„[…]perhaps also clarify some of the need for dialogue and or at least pre-structure the conversation so that the patient somehow knows a little more precisely what he actually wants to ask and then also asks specific questions“ (N_12)*  *„I deny the discharge. Because. Yes. Education was reduced to a certain extent. But what was saved in terms of the nurse's workload was then absorbed again by the documentation work” (N_17)*  *„The point was that the patients were given access. For them, for the online portal. And then. But also for the secuTrial. If you wanted to answer the questionnaires in it, and I think you got quite a lot mixed up with that“ (SN_11)* |
| **Attitude towards DiGAs**   - Advantages of DiGAs - Limitations of DiGAs - Changed attitude towards DiGAs - More suitable counseling concepts - Recommendation/prescription of levidex | The interviews revealed ambivalent opinions on digital health applications (DiGAs). The most common comment was that DiGAs could not replace personal contact with treating physicians. Concerns were expressed that patients with visual, sensory or motor impairments might have difficulties using the applications. It was also criticised that an essential part of treatment could be lost due to the lack of personal contact with carers. There was also concern that mental symptoms could not be recognised and treated in time due to the lack of personal contact, as technical applications cannot fully take over diagnostics in this area.  The advantages of DiGAs were cited as being able to provide neutral and structured information with a reliable level of evidence. The constant availability in everyday life and the associated accessibility were also emphasised. It was also mentioned that DiGAs could enable continuous support without high personnel costs due to their everyday use.  The attitude towards DiGAs has changed among the interviewees to the extent that one interviewee only became aware of the existence of these services as a result of the study. For the most part, the participants indicated a positive and open attitude towards DiGAs. Two people stated that they were still sceptical, as there was still no scientific evidence of the effectiveness of levidex. However, a large proportion of respondents continued to value personal counselling as an important factor and considered DiGAs to be merely a useful addition to the treatment spectrum.  Seven of the respondents stated that they would prescribe or recommend levidex. This was based on the positive feedback from the patients participating in the study, the desire to educate MS sufferers and to provide evidence-based information. One person stated that they would only prescribe levidex on request. The reason given was that the evidence situation was still unclear at the time of the interview. | *„Well, because I have said that I consider these lifestyle interventions to be sensible and important, because the bottom line is that they also save me time and I am talking to a well-informed patient who is not just talking about MS, which can also have a positive influence on many other illnesses throughout his life.“ (N_01)*  *„[..]And if I know that I can prescribe a health application whose contents are trustworthy and where the patient then perhaps doesn't have to search so much, but gets everything from a single source, then that's an advantage.“ (N_22)*  *„It is something that can be customised to the individual user in a certain way. It's not something fixed, so to speak. And that, I think, is the advantage. That's what digitalisation can bring. Compared to traditional media such as brochures, educational events and these things“ (N_23)*  *„Nowadays, there's a lot of talk about more interaction, video, etc. I mean, it was really well done, no question, but it was also very text-heavy somewhere“ (N_17)*  *„It is the lowest-threshold offer. When it comes to MS patients, we are talking about typical representatives of a media-trained generation and we are targeting them with the DiGAs. And in that respect it is a good instrument.“ (N_10)* |

1. **Radiologists (n=4)**

| **Categories & subcategories** | **Summary** | **Anchor examples** |
| --- | --- | --- |
| **Background & participation**   - Reasons for participation/expectations - Information about the study - Perception of the preparation for conducting the study | This category summarises the background to participation in the POWER@MS1 study as well as the perception of the information provided for the study and the preparation for the radiological procedure.  Reasons for participation among the participants were in particular the long-standing cooperation with neurologists involved in the study as well as an appropriate level of radiological effort. The objectives of the study were known to the radiologists surveyed and one person rated the approach of the study as very interesting. The interviewees stated that they had received all the necessary information (e.g. specific instructions for sequences) in advance so that the implementation could run smoothly. One person stated that they would have liked more background knowledge on the clinical relevance of the study. | *‘Because we were told this by our neurologist, ultimately. [...] We are often confronted with it by clinical colleagues. That's why it's part of our everyday life. And how I feel about it ultimately always depends a bit on the study itself, how time-consuming it is. And at first I didn't find that restrictive and that was okay for me.’ (P1_R_021)*  *‘I actually really like the idea of simply trying to pass on valid information to the patient. So I think that also has a lot to do with being self-determined, even as a patient. [...] For our part, it all worked very well. We got all the information we needed. We were able to implement the programme so wonderfully. There were specific guidelines as to what the sequences should look like.’ (P1_R_017)*  *‘We are a large radiology practice. My colleagues did that too. I wrote work instructions for the other colleagues here in the practice and that worked, so there were hardly any problems in the end.’ (P1_R_008))* |
| **Relevance of close-meshed MRIs** | This category addresses the perception of close MRI follow-up.  The radiologists rated close MRI checks alongside clinical visits as important and useful for monitoring disease activity. They recommended carrying these out beyond the annual cycle (e.g. every 6 months) and primarily without the use of contrast agents in order to prevent pathological gadolinium deposits. In the case of stable disease activity after the start of immunotherapy, one radiologist assessed an annual cycle as sufficient, but at the same time pointed out that this decision should ultimately be made by clinicians. It was also expressed that the localisation of the lesions should be taken into account when assessing the relevance of close MRI examinations. | *‘I think it's important to see what. Whether there is a certain amount of activity. Ultimately, the question is always whether you can do it purely clinically. But there are definitely differences. Some people have lesions that go unnoticed. I think it makes sense to primarily monitor them more closely, perhaps twice a year, or not always at annual intervals. [...] The fact that we don't actually use any contrast media at all now, and that the new criteria mean that there are hardly any repeated contrast media images or iv. contrast media, there is actually nothing to be said against it.’ (P1_R_008)*  *‘So I think it's relatively important, especially in the early stages, if you don't realise as a patient that something is happening, that you actually have a clinic, and that happens frequently, that you monitor this with imaging, that you also look to see where the foci are located. There are also different levels of relevance. If I now have a juxtacortical focus, yes in the grey matter and so on. So I think it's important to carry out regular close imaging checks, yes.’ (P1_R_017)* |
| **Perception of the study administration**   - Typical MRI appointment - Organisational effort - Dispatch of the CD's - Availability of free slots - Influence on daily work routine | This category summarises the perception of study administration in connection with POWER@MS1.  The respondents perceived the study administration including the organisational effort (e.g. making appointments, adjusting the devices to non-standard MRI sequences) as unproblematic, as the performance of the MRI examinations in connection with the POWER@MS1 study had no relevant influence on their usual working day. Sending the MRI findings to the study centre in Hamburg (by post on CD) was also assessed as easy to handle after a few initial difficulties. | *‘Well, the patients were registered via the neurology department and then we made an appointment here, which was then used to schedule the appointment and in close coordination and then the patient came. Yes, it was unproblematic and went well. [...] All in all, it was fine, so it didn't take much more work, or it went smoothly.’ (P1_R_001)*  *‘That's the only thing you have to get used to, so to speak, because it's simply different sequences and sometimes different directions and different levels that are ultimately used to assess progress than we do as standard. [...] It wasn't so conspicuous that I practically lost my routine, the routine technical activity. And that is ultimately decisive for our clinician. So I think it's fine to do something like that, to take part in studies, as long as you can still get on with the routine business and work together.’ (P1_R_021)*  *‘It wasn't so clear at first, because we were given these stamped envelopes and we put the findings and the CD in them and that went well afterwards. After the first three cases, it went without a hitch. There were always a few that were enquired about. You have to say that too. I don't know now, because I wasn't always with them, with the patients, but they were then sent on. But that wasn't really a problem in terms of handling.’ (P1_R_008)* |
| **MRI standard**   - Classification according to McDonald 2017 - Review of spatial dissemination - Appropriateness of MRI standard & funding - Thoughts on the spinal MRI standard | This category addressed the use of MS-specific classification standards and the perception of the appropriateness of the MRI standard used in POWER@MS1 and the funding of the MRIs performed as part of the study. In addition, thoughts on the introduction of a spinal MRI standard were summarized.  The respondents stated that they partly classify according to McDonald 2017 and check the spatial dissemination according to Swanton criteria, but in some cases also describe the MRI findings purely descriptively. There were differing opinions regarding the remuneration of the MRI examinations within the scope of the study. While some of the radiologists surveyed felt that the remuneration for MRIs was appropriate, others rated the remuneration as below average. The radiologists rated the standardization of spinal MRI imaging as sensible. In order to achieve this standardization in the future, one interviewee recommended first working on the comparability of MRI imaging in the private practice sector, as different quality criteria and frequencies are often used here.  Finally, this category summarizes the assessment of the general involvement of radiologists in the implementation of studies, retrospective feedback on participation in POWER@MS1 and suggestions for optimization.  In principle, the radiologists found it useful to be involved in studies in order to be able to exchange new findings on an interdisciplinary basis and generate data across the board. The majority of radiologists were satisfied with their participation in the POWER@MS1 study and had no suggestions for improvement. According to one interviewee, there is only potential for optimisation in the provision of information on the background and clinical relevance of the study before the start of the study. In addition, the wish was expressed that the results of the study should also be made available to the participating radiologists. | *‘It's usually the case that we have a standardized text finding or text module where the lesions typical of MS are mentioned, such as periventricular, juxtacortical, infratentorial, and this is then recorded. And sometimes this is also included in the subordinate clause, but it is more the case that we describe descriptively where the lesions are. And to be honest, there are also overlaps in the categorisations.’ (P1_R_001)*  *‘But that is generally recognised and it is a certain assessment option and, and, and. We speak the same language.’ (P1_R_008)*  *‘I think now compared to a standard knee MRI, I think we charge around €250 for private patients and a similar number of sequences and a similar amount of time. So I think it's a bit less than we would get for a standard private patient, for example.’ (P1_R_021)* |
| **Conclusion on general participation in studies & participation in POWER@MS1**   - General involvement of radiologists in studies - Retrospective feedback on participation in POWER@MS1 - Potential for optimisation | Finally, this category summarises the assessment of the general involvement of radiologists in conducting studies, retrospective feedback on participation in POWER@MS1 and suggestions for optimisation.  In principle, the radiologists found it useful to be involved in studies in order to be able to exchange new findings on an interdisciplinary basis and generate data across the board. The majority of radiologists were satisfied with their participation in the POWER@MS1 study and had no suggestions for improvement. According to one interviewee, there is only potential for optimisation in the provision of information on the background and clinical relevance of the study before the start of the study. In addition, the wish was expressed that the results of the study should also be made available to the participating radiologists. | *‘I found that interesting. Also in general, you always update yourself a bit on what's new in terms of studies, projects relating to MS monitoring or therapy. So I enjoyed that.’ (P1_R_017)*  *‘I don't have any criticism to make, so to speak, because it was really easy to handle compared to other studies.’ (P1_R_021)*  *‘Maybe a bit more background knowledge or something that would actually been nice if you were also given the results again and so on. And maybe a bit more clinical relevance of the study. I wouldn't think that would be a bad thing if you could get more information beforehand.’ (P1_R_008)* |

|  | **Baseline**  **N=234** | | | **Month 3**  **N=224** | | | **Month 6**  **N=223** | | | **Month 12**  **N=218** | | |
| --- | --- | --- | --- | --- | --- | --- | --- | --- | --- | --- | --- | --- |
|  | **levidex**  n=115 | **dexilev**  n=119 | *p-value* | **levidex**  n=111 | **dexilev**  n=113 | *p-value* | **levidex**  n=111 | **dexilev**  n=112 | *p-value* | **levidex**  n=107 | **dexilev**  n=111 | *p-value* |
| ***Please rate your knowledge about multiple sclerosis.^1^*** | 4.2 (1.7) | 4.5 (2.0) | 0.14 | 6.4 (1.5) | 6.9 (1.5) | **0.03** | 6.3 (1.7) | 6.8 (1.5) | **0.02** | 6.6 (1.7) | 7.0 (1.5) | **0.03** |
| ***I understood the information on the platform well. ^1^*** | n.e | n.e | - | 8.5 (1.6) | 8.1 (1.4) | 0.44 | 8.7(1.9) | 8.8 (1.5) | 0.78 | 8.5 (2.1) | 8.1 (2.3) | 0.25 |
| ***I found my way around the information platform easily.^1^*** | n.e | n.e | - | 8.3  (2.1) | 8.3  (1.9) | 0.94 | 8.3 (2.2) | 7.7 (2.5) | 0.10 | 7.7 (2.1) | 7.1 (2.5) | **0.04** |
| **n.e = not enquired; SD = Standard deviation**  **Items assessed on a Likert scale ranging from 0 to 10, group comparisons were conducted using independent-samples t-tests.. Statistically significant p-values (p<0.05) are highlighted in bold.**  **^1^ = VAS rating on a scale from 0 to 10, where ‘0’ is no knowledge/disagree and ‘10’ is great knowledge/agree completely *(mean/SD)*** | | | | | | | | | | | | |

**Supplement E: Evaluation of MS Knowledge, Usability, and Comprehension of Information Across Groups**
